# Phenolics from *Chrozophora oblongifolia* Aerial Parts as Inhibitors of α-Glucosidases and Advanced Glycation End Products: In-Vitro Assessment, Molecular Docking and Dynamics Studies

**DOI:** 10.3390/biology11050762

**Published:** 2022-05-17

**Authors:** Hossam M. Abdallah, Albraa T. Kashegari, Akram A. Shalabi, Khaled M. Darwish, Ali M. El-Halawany, Mardi M. Algandaby, Sabrin R. M. Ibrahim, Gamal A. Mohamed, Ashraf B. Abdel-Naim, Abdulrahman E. Koshak, Peter Proksch, Sameh S. Elhady

**Affiliations:** 1 Department of Natural Products, Faculty of Pharmacy, King Abdulaziz University, Jeddah 21589, Saudi Arabia; albraatareq@gmail.com (A.T.K.); gahussein@kau.edu.sa (G.A.M.); aekoshak@kau.edu.sa (A.E.K.); ssahmed@kau.edu.sa (S.S.E.); 2 Department of Pharmacognosy, Faculty of Pharmacy, Cairo University, Giza 11562, Egypt; akramali2010@yahoo.com (A.A.S.); ali.elhalawany@pharma.cu.edu.eg (A.M.E.-H.); 3 Department of Medicinal Chemistry, Faculty of Pharmacy, Suez Canal University, Ismailia 41522, Egypt; khaled_darwish@pharm.suez.edu.eg; 4 Department of Biological Sciences, Faculty of Science, King Abdulaziz University, Jeddah 21589, Saudi Arabia; malgandaby@kau.edu.sa; 5 Preparatory Year Program, Department of Chemistry, Batterjee Medical College, Jeddah 21442, Saudi Arabia; sabrin.ibrahim@bmc.edu.sa; 6 Department of Pharmacognosy, Faculty of Pharmacy, Assiut University, Assiut 71526, Egypt; 7 Department of Pharmacology and Toxicology, King Abdulaziz University, Jeddah 21589, Saudi Arabia; aaabdulalrahman1@kau.edu.sa; 8 Institute of Pharmaceutical Biology and Biotechnology, Heinrich-Heine-Universität Düsseldorf, Universitätsstrasse 1, 40225 Düsseldorf, Germany; proksch@uni-duesseldorf.de

**Keywords:** 1,3,6-trigalloyl glucose, α-glucosidase, diabetes, diabetic complications, molecular docking, molecular dynamics, human intestinal maltase-glucoamylase α-glucosidases

## Abstract

**Simple Summary:**

The chemical investigation of *Chrozophora oblongifolia* aerial parts resulted in the isolation of five phenolic compounds. The isolated metabolites were tested for their antioxidant and advanced glycation end-products (AGEs) formation, α-glucosidase, and lipase inhibitory activities. 1,3,6-Trigalloyl glucose exhibited the highest activity as an antioxidant and AGEs inhibitor as well as an α-glucosidase inhibitor. It showed promising binding affinity and stability towards the human intestinal maltase-glucoamylase α-glucosidases, as revealed through coupled molecular docking and dynamics studies that could encourage the utilization of this compound in the management of diabetes and its complications.

**Abstract:**

Modern life is associated with low physical activity that leads to the accumulation of fats, gaining more weight, and obesity. Accumulation of fat in the abdomen region contributes to diabetes via insulin resistance and hyperglycemia. Polyphenols are major plant constituents that exert antidiabetic activity through different mechanisms, including radicle scavenging activity, regulation of glucose uptake, and inhibition of fat and polysaccharide hydrolysis in addition to their inhibitory role regarding the formation of advanced glycation end products (AGEs). Chemical investigation of *C. oblongifolia* aerial parts resulted in the isolation of five major compounds: apeginin-7-O-β-D-glucoside (**1**), quercetin-3-O-β-D-glucuronic acid (**2**), quercetin-3-O-β-D-galacturonic acid (**3**), rutin (**4**), and 1,3,6-trigalloyl glucose (**5**). The isolated compounds were tested for their antioxidant and AGEs formation, α-glucosidase, and lipase inhibitory activities. Compound **5** revealed the highest antioxidant and AGEs inhibitory activity in bovine serum albumin (BSA)-methylglyoxal, BSA-fructose, and arginine-methylglyoxal models. Moreover, it exhibited a potent inhibitory profile on *Saccharomyces cerevisiae* α-glucosidases compared to the positive control, acarbose. Compound (**5**) further depicted promising binding affinity and stability towards the human intestinal maltase-glucoamylase α-glucosidases, which is a diabetes-related therapeutic target, through coupled molecular docking and dynamics studies. The obtained results encourage the usage of 1,3,6-trigalloyl glucose in the management of diabetes and its complications. However, detailed in-vivo studies for this compound should be performed.

## 1. Introduction

Diabetes is a complex chronic metabolic disease resulting from insulin deficiency or resistance of receptors to released hormone [1]. The disturbance of the natural balance between glucose homeostasis, proteins, and fat metabolism is the main hallmark. Diabetes incidence increases dramatically by four-fold from 2014 compared to 1980 according to World Health Organization [2]. Diabetes is considered the seventh cause of mortality worldwide according to USA National Center for Health Statistics [3].

Protein glycation represents one of the critical pathways involved in the progress of diabetic complications. Chronic hyperglycemia triggers postsynaptic protein alteration through a series of reactions called Maillard reactions [4]. Non-enzymatic glycosylation of amino groups of essential proteins with the carbonyl group of sugars drives the formation of complex products called advanced glycation end products (AGEs) [5]. AGEs may exert a pivotal role in the formation of metabolic memory in diabetic complications with accumulating evidence to link glycation and insulin resistance [6,7]. The glycation process and AGEs formation are usually associated with increased production of reactive oxygen species (ROS) [8]. Unfortunately, synthetic antidiabetic drugs might exert many side effects [2,5]. In this regard, there is a scientific interest to identify natural AGEs inhibitors with a good antioxidant potential to serve as a safe alternative approach for treating diabetic complications.

Lifestyle and Medicinal plants could be helpful in the management of diabetic complications. The regular practice of physical activity results in greater energy expenditure and a lower risk of diabetic complications. Moreover, a plant-based diet that is based on using definite amounts of whole-grain cereals, legumes, nuts, and olive oil will be supportive. Legumes and cereals are able to reduce postprandial glucose levels by decreasing carbohydrate absorption due to their high complex carbohydrates and fiber contents [9]. Moreover, many medicinal plants are known for activity in diabetic complications, like green tea, *Garcinia cambogia*, *Nigella sativa*, *Hibiscus sabdariffa*, *Caralluma fimbriata* [10], *C. tuberculata* [11], and *C. hexagona* [12].

The spurge family, Euphorbiaceae, is a large flowering plant family with 300 genera and about 7500 species that has different traditional uses [13]. *Chrozophora* genus belongs to the Euphorbiaceae family, which contains seven to eight species, most of which are monoecious herbs. This species is found in the Mediterranean regions, as well as in Pakistan, India, and West Africa. The genus is characterized by the presence of phenolic compounds [14] that showed different biological activities including anti-microbial [15], α-glucosidase inhibitory [16], anti-inflammatory [17], and osteoprotective [18]. In continuation of our work on diabetic complications [4,19,20,21] and based on the few data concerning active constituents of *C. oblongifolia* (L.) A. Juss; it was deemed important to carry out a biological investigation of its phenolic constituents and assess their effect on diabetic complications.

## 2. Materials and Methods

### 2.1. Plant Material

The flowering aerial parts of *C. oblongifolia* were collected from Al-Hadda road, Altaif governorate, Saudi Arabia, in April 2018. Authentication of the plant was established by Dr. Emad Al-Sharif, Associate Professor of plant ecology, Dept. of Biology, Faculty of Science and Arts, Khulais, King Abdulaziz University, Kingdom of Saudi Arabia. A voucher specimen (Reg. No. CO-1080) is kept in the herbarium of the Department of Natural Products and Alternative Medicine, Faculty of Pharmacy, King Abdulaziz University, KSA.

### 2.2. Extraction and Isolation

One Kg of air-dried powdered flowering aerial parts of *C. oblongifolia* was extracted with methanol (MeOH) (4 × 5 L) at room temperature using Ultraturrax, till exhaustion. The combined extracts were filtered and concentrated to give a dark brown residue of 100 g. The residue was suspended in 500 mL distilled water and extracted with chloroform (CHCl_3_) (4 × 500 mL) to give 64 g of dry extract. The concentrated remaining mother liquor was fractionated on the Diaion HP-20 column, eluted with water (H_2_O) (1 L), and subsequently eluted with MeOH/H_2_O (50%, 2.4 L) and MeOH (100%, 2.7 L), respectively. The eluates were evaporated under vacuum to give 9.7 g (fraction A), 18 g (fraction B), and 7.8 g (fraction C). Fraction A contained free sugars and exhibited no polyphenol character, by tracing on TLC using aluminum chloride (AlCl_3_) and ferric chloride (FeCl_3_). Fraction C was fractionated over SiO_2_ CC (50 × 5 cm, 180 g) using CHCl_3_:MeOH gradient elution to give three major fractions, I, II, and III. Fraction I (0.5 g) was fractionated on Sephadex LH-20 column chromatography (CC) with MeOH as an eluent to afford compound **1** (50 mg). Fraction II (1.5 g) was chromatographed on SiO_2_100 C_18_– reversed-phase CC using MeOH:H_2_O (3:7) as an eluent to afford compound **2** (40 mg). Fraction III (2 g) was subjected to repeated chromatographic fractionations on SiO_2_100 C_18_–reversed-phase CC using MeOH:H_2_O (3:7) as an eluent to yield compound **3** (20 mg). Fraction B was fractionated over SiO_2_ CC chromatography (50 × 5 cm, 180 g) using CHCl_3_:MeOH gradient elution to give two major, I and II. Fraction I (0.5 g) was chromatographed on SiO_2_ 100 C_18_ reversed-phase CC using MeOH:H_2_O, 4:6 as an eluent to afford compound **4** (50 mg). Fraction II (1.5 g) was submitted to SiO_2_ 100 C_18_-reversed-phase CC using MeOH:H_2_O (3:7) as an eluent to give compound **5** (40 mg).

### 2.3. Characterization of Isolated Compounds

Structure elucidation of isolated compounds was performed by 1D and 2D NMR on Bruker Avance DRX 850 MHz spectrometers (Bruker BioSpin/Billerica/MA/USA).

### 2.4. Biological Evaluation

#### 2.4.1. Inhibition of α-Glucosidase

α-Glucosidase from *Saccharomyces cerevisiae* (Sigma-Aldrich, St. Louis, MO, USA cat#G003) was used in this assay. Solutions of the different compounds were prepared in 1000 µM final concentrations in dimethyl sulfoxide (DMSO). Compounds that exceeded 50% inhibition at this concentration were serially diluted to determine their half-maximal inhibitory concentration (IC_50_). As a positive control, acarbose was prepared in MeOH at a concentration of 1 mM. α-Glucosidase inhibition was assessed colorimetrically using p-nitrophenyl-α-D-glucopyranoside as the substrate (pNPG, Sigma-Aldrich, St. Louis, MO, USA cat#N1377) as previously reported with slight modifications [22].

Briefly, 25 μL of samples/blank were incubated for 10 min at 37 °C with 50 μL of α-glucosidase (0.6 U/mL) in phosphate buffer (0.1 M, pH 7) in 96-microwell plates. The mixture was then incubated at 37 °C for 5 min with 25 μL of 3 mM pNPG in phosphate buffer (pH 7). A microplate reader (TECAN, Männedorf, Switzerland) was used to measure p-nitrophenol release from pNPG substrate at 405 nm to determine enzyme activity. Then, % α-glucosidase inhibition was estimated.

#### 2.4.2. Inhibition of Pancreatic Lipase

The inhibition of pancreatic lipase activity was assessed using *p*-nitrophenyl dodecanoate (*p*-NPD, Sigma-Aldrich, St. Louis, MO, USA cat#N0252) as substrate and porcine pancreatic lipase type II (PPL, sigma Aldrich, cat#L3126) which was described previously [23] with slight modification. Solutions of the provided compounds were prepared in final concentrations of 50 µM in 5% DMSO. Orlistat was used as positive control and was prepared in assay buffer. In a Tris-HCl buffer (100 mM, pH 8); 50 μL of PPL (1 mg/mL) was incubated with 25 μL of samples/blank for 10 min at 37 °C. Then, 10 μL of solution *p*-NPD (10 mM, in isopropanol) was added followed by dilution of the final volume to 200 μL with Tris-HCl buffer. The mixture was incubated for 20 min at 37 °C and absorbance was then measured at 405 nm on a TECAN microplate reader (Männedorf, Switzerland). After that, the percentage of lipase inhibition was assessed (Supplementary Table S1 and Figure S1).

#### 2.4.3. Inhibition of the Formation of Advanced Glycation End Products (AGEs)

The isolated compounds were prepared at final concentrations of 500 µM in DMSO in the BSA-fructose assay, and 1000 µM in all other AGEs assays. Compounds that exceeded 50% inhibition at this concentration were serially diluted to determine their IC_50_. All tests were conducted using quercetin (positive control).

#### BSA-Fructose Assay

According to the previous report [8], the inhibition test for AGE formation was assessed using the BSA-fructose model. Briefly, BSA (50 mg/mL) and fructose (1.25 M) (in 200 mM phosphate buffer, pH 7.4, with 0.02% sodium azide) were incubated with Sample/blank in the dark for 72 h at 37 °C, (1:1:1 by volume). On a microplate reader (Fluostar Omega BMG Labtech, Ortenberg, Germany), fluorescent AGEs were detected using excitation and emission wavelengths of 355 nm and 460 nm, respectively. Triplicates of all experiments were performed, and the percentage of AGEs inhibition was calculated.

#### Arginine-Methylglyoxal Assay

The determination of the inhibitory effect of the isolated compounds on the formation of AGEs in the arginine methylglyoxal model was used as described previously with slight modification [8]. Briefly, in black 96-microwell plates, a sample was incubated for 72 h at 37 °C in the dark with methylglyoxal (53.3 mM, in distilled water) and 106.6 mM arginine (in 200 mM phosphate buffer, pH 7.4, with 0.02% sodium azide), (1:1:1 *v*/*v*/*v*). On a microplate reader (Fluostar Omega/BMG Labtech/Germany), fluorescent AGEs were detected using excitation and emission wavelengths of 355 nm and 460 nm, respectively. Triplicates of all experiments were performed.

#### BSA-Methylglyoxal Assay

AGEs inhibitory activity of the isolated compounds was determined according to the reported procedures [8] with slight modification. Briefly, in black 96-microwell plates, samples were incubated for 72 h at 37 °C in the dark with bovine serum albumin (BSA, 50 mg/mL) (in 200 mM phosphate buffer, pH 7.4, with 0.02% sodium azide) and methyl-glyoxal (53.3 mM, in distilled water), (1:1:1 *v*/*v*/*v*). On a microplate reader (Fluostar Omega/BMG Labtech/Germany), fluorescent AGEs were detected using excitation and emission wavelengths of 355 nm and 460 nm, respectively. Triplicates of all experiments were performed.

#### 2.4.4. Antioxidant Activity

##### 2,2-diphenyl-1-picrylhydrazyl (DPPH) Assay

Isolated compounds were prepared in final concentrations of 1000 µM in DMSO. Compounds that exceeded 50% inhibition at this concentration were serially diluted to determine their IC_50_. Ascorbic acid was used as a positive control, prepared in a concentration of 100 µM in water from which six concentrations were prepared to calculate its IC_50_. The method is based on the reduction of free radical of 2,2-diphenyl-1-picrylhydrazyl (DPPH) [22]. Briefly, in a 96-well plate, 20 μL of the isolated compound was incubated for 20 min in the absence of light with 180 μL of DPPH reagent (100 μM). The reduction of intensity in the color of DPPH was assessed on a microplate reader (TECAN, Männedorf, Switzerland) at 540 nm (Supplementary Table S2).

##### 2,2′-azino-bis (3-ethylbenzothiazoline-6-sulfonic acid) (ABTS) Assay

DMSO was used for the preparation of 0.5 mg/mL concentrations of isolated compounds, meanwhile, the positive control (ascorbic acid) was used to prepare a stock solution of 1000 µM in water; from which seven concentrations were prepared including 7.812, 15.625, 31.25, 62.5, 125, 250 and 500 µM to construct the concentration-response curve (Supplementary Data Figure S2). The activity of the tested compounds to scavenge the free radicals of ABTS (2,2′-azino-bis (3-ethylbenzothiazoline-6-sulfonic acid)) was assessed according to the methodology reported previously [20] with slight modifications.

Five milliliters of aqueous solution (7 mM) of ABTS was mixed with 88 µL potassium persulfate (140 mM) and the mixture was kept in the dark for 16 h to produce ABTS radical cation (ABTS^•+^). Then methanol was added to the reaction mixture to dilute the formed ABTS^•+^ solution to an absorbance of 0.700 (1:50) at 690 nm.

Ten microliters of the tested compound were incubated with 190 μL of ABTS^•+^ solution in a 96-well plate for thirty minutes in the dark and the absorbance was measured later using a microplate reader (TECAN, Männedorf, Switzerland) at 690 nm. Data are represented as means (*n* = 3) ± SD and the antioxidant effect of the tested compounds was calculated as µM Ascorbic acid equivalents by substitution in the linear regression equation (Supplementary Figure S2).

##### Ferric Reducing Antioxidant Power (FRAP) Assay

Isolated compounds were dissolved in DMSO to prepare a 0.5 mg/mL concentration, meanwhile, the positive control (ascorbic acid) was used to prepare a stock solution of 1000 µM in water; from which seven concentrations were prepared including 15.625, 31.25, 62.5, 125, 250 and 500 µM to construct the dose-response curve. The method of the assay depends on the reduction of Fe^3+^ to Fe^2+^, which is chelated by 2,4,6-tris(2-pyridyl)-s-triazine (TPTZ) to form Fe^2+^-TPTZ complex which is known as FRAP (ferric reducing antioxidant power) [1]. Briefly, 190 μL of FRAP reagent (300 mM sodium acetate buffer pH 3.6, 10 mM TPTZ, and 20 mM ferric chloride, (10:1:1)) was incubated in the dark with 10 μL of the isolated compound in a 96-well plate for 30 min at 37 °C and absorbance was determined at 620 nm using microplate reader (Tecan, San Jose, CA, USA). Data are represented as means (*n* = 3) ± SD and the antioxidant effect of the extract was calculated as µM Ascorbic acid equivalents by substitution in the linear regression equation (Supplementary Figure S3).

##### Metal Chelation Assay

Isolated compounds were dissolved in DMSO to prepare a concentration of 0.5 mg/mL, meanwhile, the positive control (EDTA) was used to prepare a stock solution of 100 µM in water; from which six concentrations were prepared including 5, 10, 20, 40, 50 and 80 µM to construct the dose-response curve. The assay was carried out as previously reported [24], with minor modifications. Briefly, in 96 wells plate (*n* = 6) 20 µL of the freshly prepared ferrous sulfate (0.3 mM) was mixed with 50 µL of the tested compound and incubated with 30 µL of ferrozine (0.8 mM) for 10 min at room temperature. Finally, reduction in color intensity was assessed at 540 nm using a microplate reader (Tecan, San Jose, CA, USA). Data are represented as means (*n* = 3) ± SD and the antioxidant effect of the extracts was calculated as µM EDTA equivalents by substitution in the linear regression equation (Supplementary Figure S4).

##### Oxygen Radical Absorbance Capacity (ORAC) Assay

Isolated compounds were dissolved in DMSO to prepare a concentration of 0.25 mg/mL, meanwhile, the positive control (Trolox) was used to prepare a stock solution of 1 mM in MeOH; from which nine concentrations were prepared including 400, 300, 200, 150, 100, 75, 50, 25, and 12.5 µM to construct the dose-response curve. The assay was carried out as previously reported [25], with minor modifications. Briefly, 12.5 µL of the prepared sample(s) were incubated with 75 µL fluoresceine (10 nM) for 30 min at 37 °C. Measuring fluorescence was performed (520 EM, 485 EX, nm) for 3 cycles (90 s cycle time) for background measurement (Supplementary Figure S5). Subsequently, to each well, freshly-made AAPH (2,2′-azobis(2-amidinopropane) dihydrochloride) (12.5 µL, 240 mM) was immediately added. Measuring fluorescence (520 EM, 485 EX, nm) was maintained (90 s × 85 cycles) for 2.5 h (Supplementary Figure S5). The antioxidant potential (µM trolox equivalents) was estimated by substituting in the linear-regression equation.

#### 2.4.5. Data Analysis

All IC_50_ data were depicted as means ± SE and analyzed utilizing Microsoft_Excel^®^ and IC_50_s were estimated by Graph-pad-Prism 8^®^. One-way ANOVA followed by Tukey’s test was utilized for significant differences between means.

### 2.5. Ligand-Target Preparation and Molecular Docking Analysis

Atomistic models of the designated gallotannin derivative and reference antagonist of the human intestinal maltase-glucoamylase α-glucosidase enzyme (*h*MGAM) were constructed via the builder module within the MOE2019.01 software package (Chemical Computing Group^TM^, Montreal, QC, Canada). Ligands were constructed within a 3D-representative fashion based on PubChem-deposited SMILES line annotations. Adopting the 2000 step-conjugate-gradient approach, ligands were energy-minimized down to 1 × 10^−3^ Kcal/mol/Å^2^ RMS-based gradient convergence under MMFF94s and MMFF94s-modified forcefields [26,27,28].

For the biological target, we retrieved the deposited 3D-crystallographic files at the RCSB-Protein Data Bank for the atomic structure of *h*MGAM (PDB entry: 2QMJ). Files were structurally prepared through 3D-protonation at pH 7.4, 300 K, and 100 mM salt solution, as well as auto-corrections of bond connectivity, partial charge, and atom library signatures. Missed protein loops were modeled through MOE_loop_modeler. Binding sites were identified using the MOE_Alpha_site_Finder and refined to include the pocket’s key residues that are reported within the literature [29]. Lining residues included; Arg202, Asp203, Thr204, Thr205, Pro206, Asn207, Asn209, Thr211, Tyr214, Arg298, Tyr299, Asp327, Ile328, Ile364, Trp441, Asp443, Met444, Ser448, Arg526, Trp539, Gly541, Asp542, Asp571, Phe575, Ala576, Leu577, Arg598, His600, Gly602, Gln603, Phe605, Val405, Trp406, Ser448, Phe450, Leu473, and Asp474.

Guided by a triangular matcher, several ligand conformations were generated and initially ranked via London_dG scoring. Subsequently, a refinement step proceeded where retained top-scored poses were rescored through a Generalized Born-solvation-VI/Weighed Surface-Area_dG (GBVI/WSA_dG)-directed protein residue-tethered minimization step for providing the final ligand/target binding modes [30]. This final score was based on electrostatics of explicit solvation, currently assigned partial charge, the surface area of exposure, and Coulomb’s electrostatics by protein-ligand van der Waals scoring [31,32]. Significant docking energy scores (kCal/mol), 2.0 Å root-mean-square deviation (RMSD) cut-off relative to co-crystalline ligand superimposition, as well as significant contacts with reported important pocket-binding residues were all considered during the selection of the best docking pose. Visualization and ligand-target binding analysis were done using PyMol_V2.0.6 (Schrödinger^TM^, New York, NY, USA) [33].

### 2.6. Molecular Dynamics (MD) Simulations

Conformational stability, protein contacts, and predicted binding scores of ligand-*h*MGAM complexes, of both investigated gallotannin derivative as well as co-crystalline ligand antagonist, were explored through 200 ns explicit MD simulations using GROMACS-2019 under CHARMM36m.forcefield for protein [34] and CHARMM-General_forcefield program for ligand modeling [35]. A periodic box of TIP3P solvent was added around the protein ensuring at least a 10 Å distance between the protein and the box [36]. Standard ionization states of *h*MGAM amino acids were assigned at pH 7.4 keeping the whole system neutralized through added potassium and chloride ions [37]. Each system was individually minimized (0.005 ns; steepest-descent method), double equilibrated (0.1 ns, 303.15 K at NVT ensemble; then 0.1 ns, 303.15 K, 1 atm. P at NPT ensemble) before the MD simulation production was conducted for subsequent 200 ns runs under NPT ensemble and Particle-Mesh Ewald protocol for modeling long-ranged electrostatic contacts [38]. linear constraint LINCS was applied to maintain covalent bond distances at 2 fs integration time-stepped sizes [39], while non-bonded interactions were truncated via the Verlet scheme at 10 Å cut-offs [40]. Stability analysis of the simulated models was performed through root-mean-square deviation (RMSD) and difference RMS fluctuation (ΔRMSF) analysis while the latter was estimated for each ligand-bound protein (*holo*) in relation to its unliganded (*apo*) state (PDB entry: 2QLY; 2.00 Å atomic resolution), where ΔRMSF = RMSF_(apo−holo)_. Finally, Molecular Mechanics/Poisson-Boltzmann Surface Area (MM/PBSA) was applied for binding-free energy calculations as well as for estimating the residue-wise energy contributions [41]. Ligand-protein conformational analysis was represented and analyzed using the PyMol_V2.0.6 software (Schrödinger^TM^, New York, NY, USA).

## 3. Results and Discussion

Chemical investigation of *C. oblongifolia* resulted in the isolation of five major compounds (Figure 1) that were identified as: apeginin-7-O-β-D-glucoside (**1**), quercetin-3-O-β-D-glucuronic acid (**2**), quercetin-3-O-β-D-galacturonic acid (**3**), rutin (**4**), and 1,3,6-trigalloyl glucose (**5**). The identity of isolated compounds was assessed by different spectroscopic methods including one- and two-dimensional NMR (Supplementary Figures S6–S23).

### 3.1. Inhibition of α-Glucosidase

**Figure 2:**
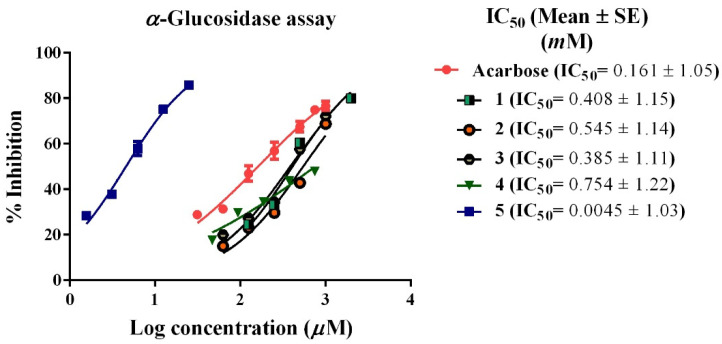
α-Glucosidase inhibitory effect of the isolated compounds (**1–5**) from *C. oblongifolia* and acarbose.

α-Glucosidase is a membrane-bound enzyme of the small intestine that represents the key enzyme in carbohydrates metabolism. Enzyme inhibition is the main target for the treatment of type II diabetes and can significantly attenuate the elevated glucose level especially postprandial blood glucose [22]. In the present study, acarbose inhibited α-glucosidase at IC_50_ of 0.161 mM. Compound **5** showed the most potent inhibition of the enzyme with IC_50_ of 0.0045 mM with a more potent inhibitory effect than acarbose. Other compounds revealed inhibitory effect on α-glucosidase in the following order **3** > **1** > **2** > **4** with IC_50_ of 0.385, 0.408, 0.545, and 0.754 mM, respectively (Figure 2).

### 3.2. Inhibition of Pancreatic Lipase

Orlistat inhibited pancreatic lipase with an IC_50_ of 0.70 μM, while all tested compounds did not show significant inhibition till 50 μM concentration (Supplementary Figure S1 and Table S1).

### 3.3. Inhibition of the Formation of Advanced Glycation End Products (AGEs)

Evaluation of cell-free in-vitro anti-glycation activity was established using three models viz: BSA-fructose, BSA-methylglyoxal, and arginine-methylglyoxal assays. Tight control of intracellular glucose concentration plays an important role in the pathogenesis of microvascular diabetes complications. The overproduction of ROS by mitochondrial stimulates the formation of AGEs as a piece of the puzzle [42].

Indeed, the generation of AGEs was established in three stages; in an early stage, a series of cascade reactions between the glucose carbonyl group and the terminal α-amino-groups of proteins produce an unstable Schiff’s base. In an intermediate stage, intermolecular rearrangements of Schiff’s base through the Maillard reaction form more stable Amadori products. In the late stage, Amadori products undergo oxidative modifications of reactive dicarbonyl compounds as glyoxal or methylglyoxal to yield irreversible AGEs compounds [43,44].

The reactive metabolite (methylglyoxal, MGO) is the permeant precursor of AGEs that are directly associated with neurodegenerative diseases and diabetic complications [45]. Regarding the glycation process, MGO reacts with different protein residues such as arginine, lysine, and cysteine to generate irreversible AGEs [42].

BSA was selected as the model protein to mimic human serum albumin. The fluorescence intensity indicated the formation of AGEs in all BSA models. The inhibition of BSA glycation prevents the cascade reactions that yield AGEs [8].

**Figure 3:**
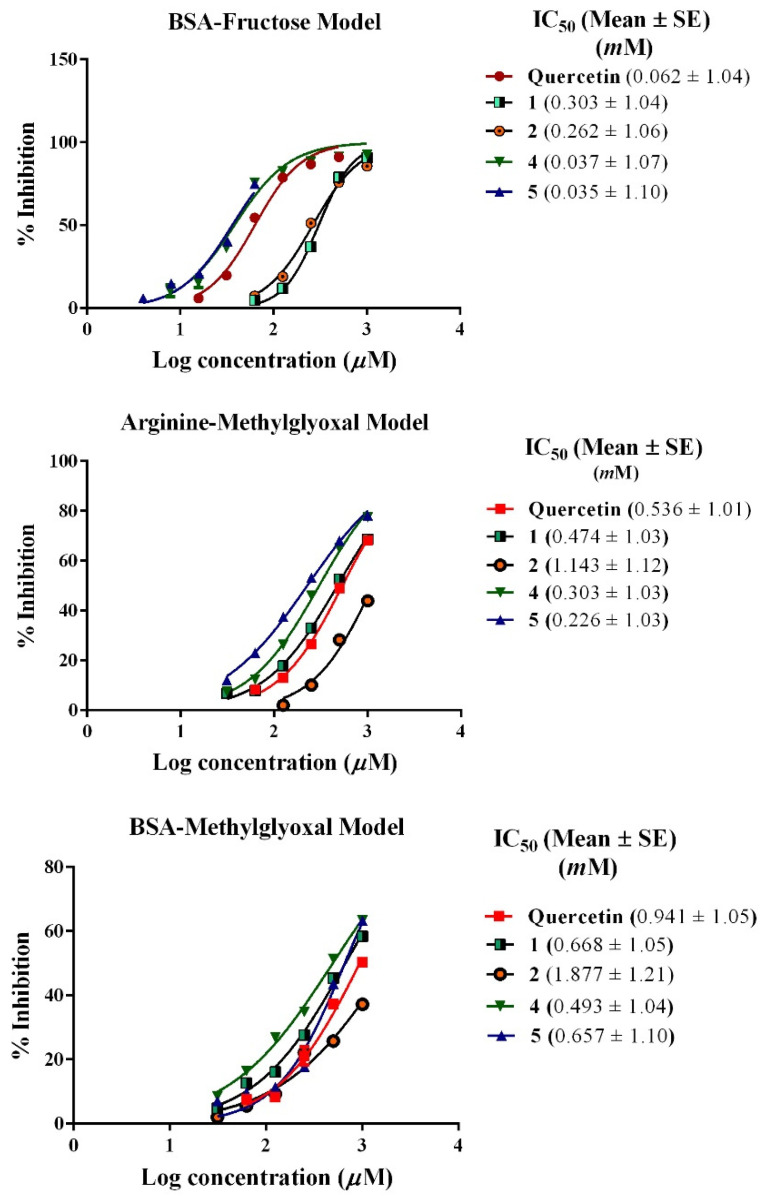
Inhibitory effect of the isolated compounds (**1**–**5**) from *C. oblongifolia* and quercetin on advanced glycation end products in the BSA-fructose, arginine-methylglyoxal model, and BSA-methylglyoxal models.

In the BSA-fructose model compound, **5** showed potent inhibition at IC_50_ of 0.035 mM, followed by compound **4** with an IC_50_ of 0.037 mM. Whilst compounds **1** and **2** significantly showed lower inhibition (IC_50_s 0.303 and 0.262 mM, respectively). Meanwhile, compound **3** showed an inhibitory effect (IC_50_ of 0.500 mM) compared to quercetin (IC_50_ at 0.062 mM) (Figure 3).

On the other hand, in the arginine-methylglyoxal assay, compound **5** showed the highest activity with an IC_50_ of 0.226 mM, followed by compounds **4**, **1**, and **2** (IC_50_ of 0.303, 0.474, and 1.143 mM, respectively) compared to quercetin (IC_50_ 0.536 mM). Compound **3** did not reveal any inhibitory effect (IC_50_ > 1.200 mM) (Figure 3).

Finally, in the BSA-methoxyglyoxal assay, quercetin revealed inhibitory IC_50_ of 0.941 mM, while compounds **5**, **4**, and **1** had IC_50_ at 0.657, 0.493, and 0.668 mM, respectively. Compound **2** showed the lowest inhibition at 1.877 mM and like the BSA fructose assay, compound **3** did not reveal any inhibitory effect with IC_50_ > 1.200 mM (Figure 3).

### 3.4. Antioxidant Activity

**Figure 4:**
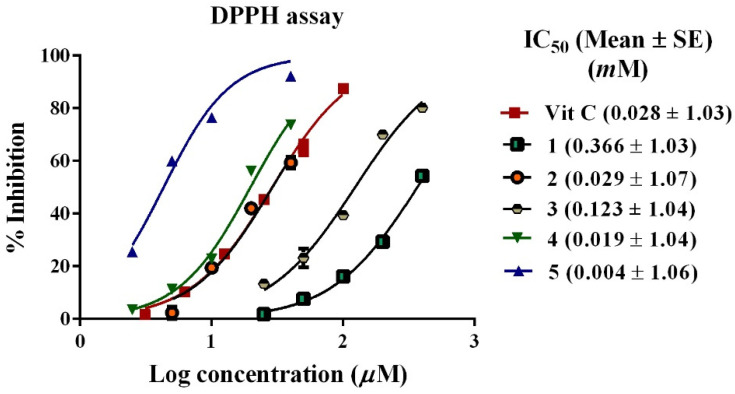
Inhibition of DPPH free radicle by isolated compounds (**1–5**) from *C. oblongifolia* and vitamin C.

**Figure 5:**
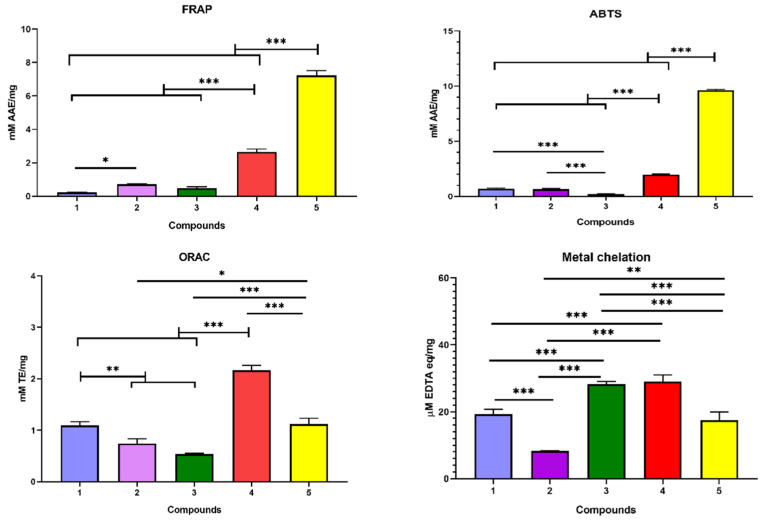
Antioxidant activity (ABTS, FRAP, ORAC and metal chelation) of isolated compounds **1**–**5** from *C. oblongifolia*. *** Significantly different at *p* < 0.0001, ** significantly different at *p* < 0.001, * significantly different at *p* < 0.05.

For assessment of free radical scavenging activity of isolated compounds, at least more than two different methods are highly recommended [46]. In the current study, five different assays (DPPH, ABTS, FRAP, metal chelation, and ORAC) have been established utilizing different mechanisms to assess more realistic measurements [47]. They were established for measuring the total antioxidant capacity (TAC) through a significantly different and specific chemical reaction. The results of the antioxidant activity of different compounds are shown in Figure 4 and Figure 5.

In the DPPH assay, ascorbic acid showed IC_50_ of 0.028 mM. Compound **5** possessed the most potent inhibition of DPPH radical with an IC_50_ of 0.004 mM, followed by compounds **4** and **2** with IC_50_ of 0.019, and 0.029 mM, respectively. While compounds **3** and **1** revealed much lower inhibition at 0.123 and 0.366 mM, respectively (Figure 4).

Generally, preventive antioxidants are enzyme-based reactions, such as catalase, peroxidase, and superoxide dismutase, which suppress ROS. On the other side, chain-breaking antioxidants are compounds that are able to scavenge tissue ROS and break the reaction chain like vitamin E, vitamin C, and phenolic compounds [25,47]. The mechanism underlying the function of chain-breaking antioxidants can be explained based on hydrogen atom transfer (HAT), single electron transfer (SET), or the combination of both HAT and SET mechanisms.

DPPH assay depends on both SET or HAT while ABTS depends only on SET [25]. Consequently, the ABTS assay may be considered a confirmatory assay to the DPPH assay. However, ABTS has an advantage over DPPH since it is pH-independent and is applicable for antioxidants of lipophilic or hydrophilic nature [25].

Unlike DPPH, the FRAP assay is a non-specific redox colorimetric assay based on the ability of the antioxidant sample to donate electrons to reduce a colorless Fe^3+^-TPTZ complex to an intense blue Fe^2+^-TPTZ complex under acidic conditions [48]. In the current study, unlike the ABTS inhibition assay, compounds **5** and **4** revealed the highest activity with 7.23 and 2.66 mM AAE/mg of the compounds. On the other hand, compounds **2**, **3**, and **1** showed lower activity at 0.73, 0.49, and 0.23 mM AAE/mg compound. Although both ABTS and FRAP are based on the SET mechanism, FRAP can measure only the reducing power of samples [48,49].

Conversely, the ORAC assay measures the capacity of hydrophilic antioxidants to capture peroxyl radicals depending on the hydrogen atom transfer (HAT) mechanism [46]. Compound **4** showed the highest activity in the ORAC assay revealing a 2.16 mM TE/mg compound. Compounds **5** and **1** revealed significant activity with 1.11- and 1.09-mM TE. Finally compounds **2** and **3** showed the lowest activity at 0.74- and 0.54-mM TE, respectively.

Finally, metal chelation is based on the capacity of antioxidant compounds to compete with ferrozine for Fe^2+^ ion in a process called the Fenton reaction. Interestingly, as illustrated in Figure 5; compounds **5**, **4**, **1**, and **3** showed high activity with 17.52, 28.96, 19.26, and 28.26 μM EDTA eq./mg compound, respectively. While compound **2** showed the lowest activity at 8.29 μM EDTA eq./mg.

Antioxidant activity plays a pivotal role to maintain cellular physiology by scavenging ROS and preventing its damaging capacity [50]. In terms of biological activity, the generation of free radicals triggers the spontaneous production of non-enzymatic AGEs due to rearrangements of Amadori products.

The aforementioned data revealed that compound **5** is the most potent with regard to all observed activities. Compound **5** revealed more potent inhibition on α-glucosidases than acarbose. Nevertheless, acarbose was renowned to be more efficient on human α-glucosidase than other sources of α-glucosidases such as *Saccharomyces*, utilized in this assay, which necessitates comparing compound **5** and acarbose on human glucosidase through docking and molecular dynamic studies. Furthermore, compound **5** did not show any inhibitory effect on pancreatic lipase which could eliminate any possibility of non-specific action of this compound on enzymes such as precipitation or denaturation.

### 3.5. Molecular Docking Analysis

**Figure 6:**
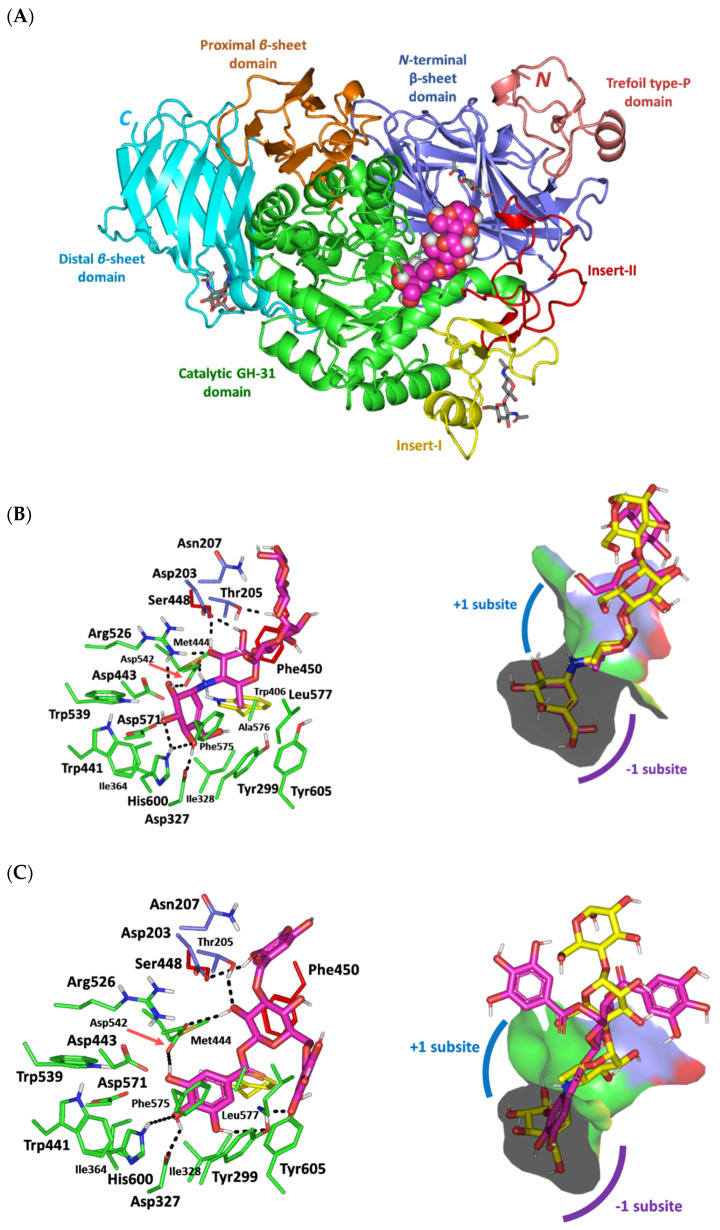
Architecture of the human intestinal maltase-glucoamylase α-glucosidase enzyme (hMGAM; PDB entry: 2QMJ) in complex with the co-crystalline and docked ligands. (**A**) Cartoon representation of the hMGAM in complex with co-crystalline ligand (magenta spheres)/glycan chains (grey sticks) showing the individual structural domains in different colors as follows; N-terminal trefoil P-type domain (deep salmon; residue range 1–51), β-sandwich sheet domain (slate blue; residue range 52–269), [α/β]_8_-barrel catalytic domain (green; residue range 270–651), with insert-I (yellow; residue range 367–416) and insert-II (red; residue range 447–492), C-terminal proximal domain (orange; residue range 652–730), and distal domain (cyan; residue range 731–868). Left panels of (**B**,**C**) are the predicted binding modes of the redocked acarbose and *C. oblongifolia* isolated 1,3,6-trigalloylglucose (magenta sticks), respectively, at the hMGAM shallow substrate-binding site. The protein target is represented as a cartoon, while as, only residues located within a 4 Å radius of bound ligand are displayed as lines, labeled with sequence number, and colored according to their structural domain location. Polar interactions, represented as hydrogen bonds, are illustrated as black dashed lines. Right panels of (**B**,**C**) are the overlay of each docked ligand (magenta sticks) in relation to the co-crystallized ligand (yellow sticks) within the hMGAM substrate-binding shown in surface representation. The −1 and +1 carbohydrate subsites are depicted with purple and blue arcs, respectively.

From a therapeutic standpoint, the above profound activity of the *C. oblongifolia*-isolated 1,3,6-trigalloylglucose (**5**) on *Saccharomyces* α-glucosidase prompted the further evaluation of the compound’s affinity towards human α-glucosidase through comprehensive in-silico study. Note that acarbose has been reported to be more effective on human α-glucosidase than α-glucosidases from other sources, such as *Saccharomyces* [51]. In this regard, the molecular docking affinity of this top-active compound was investigated against the crystalline human intestinal maltase-glucoamylase α-glucosidase enzyme (hMGAM; PDB entry: 2QMJ), in comparison with the canonical small molecule inhibitor, acarbose [52]. The adopted 101.02 kDa biological target is a monomer-A1 hydrolase glycoprotein solved at 1.90 Å atomic resolution in a complex with different glycans in addition to acarbose. The overall architecture of this α-glucosidase enzyme (Figure 6A) is of a trefoil P-type domain at the N-terminus which is then followed by a β-sandwich sheet domain and [α/β]_8_-barrel catalytic domain bearing two inserts (insert-I and -II) protruding after the β3 and β4-strands, respectively. At the hMGAM carboxy terminus, the structure ends with subsequent proximal and distal domains having distinct β-sandwich topologies comparable to the other homologous glycoside hydrolase (GH)-31 family [51,53,54,55]. The substrate-binding pocket is formed mainly by the [α/β]_8_-barrel catalytic residues, in addition to the N-terminus loop (residue range 200–217) as well as portions of catalytic inserts-I and -II near the opening of [α/β]_8_-barrel catalytic domain.

The non-cleavable crystalline ligand, acarbose, shows favored anchoring at the hMGAM active-site pocket via its two initial rings, the acarvosine unit, being positioned at the non-reducing terminal of this pseudo-α-1,4-tetrasaccharide (Figure 6B). In the X-ray structure, this acarvosine scaffold exhibited extended polar interactions with the target pocket residues at the −1 and +1 carbohydrate subsites including Asp203, Asp327, Arg526, Asp542, and His600 sidechains. Water-bridge hydrogen bonding was also depicted for the acarvosine moiety towards Asp443 and Asp571. Based on mutagenesis, substrate trapping studies, and sequence alignment with GH-31 protein members, the Asp443 and Asp542 were identified as the respective catalytic nucleophile and acid/base catalysis residue, making them the key ligand-binding amino acids for halting the hydrolase catalytic machinery [54]. The aglycone valienamine, the first ring of acarbose, adopted a ^2^H_3_-half-chair conformational structure as being lodged within the −1 subsite. This depicted orientation has directed the non-hydrolyzable inter-glycosidic nitrogen atom towards the catalytic center for hydrogen bond pairing with the Asp542 sidechain. The stability of acarbose at +1 subsite was mediated via the ligand’s C2 and C3 hydroxyl groups through polar interactions with Arg526, Asp542, and Asp203, where the last residue originated from the N-terminal loop at the β-sheet domain. Notably, loop residues at such β-sheet domain exhibited invariable interactions with several carbohydrate ligands within all available GH-31 crystalline structures [51,53,54,55,56]. Unlike the acarvosine scaffold, the acarbose’s two maltose moieties at +2 and +3 carbohydrate subunits showed limited polar interactions with the lining residues. The stability of these glycon scaffolds was rather mediated through the crystal lattice packing, hydrogen-bond pairing between the terminal maltose ring and Thr205/Asn207, as well as water-mediated binding with penultimate maltose and Tyr605 at the pocket’s rim. Further stabilizing interactions are provided via the hydrophobic contacts with Tyr299, Ile328, Ile364, Trp406, Trp441, Phe450, Trp539, Phe575, Ala576, Leu577, and Tyr605.

Redocking acarbose into the hMGAM substrate-binding site was performed through the rigid docking protocol since the superposition correlation analysis between the protein’s apo- and holo-states illustrated insignificant protein conformational changes (RMSD = 0.1 Å), either locally or globally. The latter suggested an irrelevant impact of local ligand induced-fitting on hMGAM’s holo-structure, at least within the macromolecular crystalline states [57]. This could be also the reason for the surface located shallow nature of the *h*MGAM substrate-binding pocket with possible accommodation of two carbohydrate subunits [51,52,53]. Findings from the acarbose redocking analysis revealed good docking binding energy (−6.8 kCal/mol) and low RMSD (1.5 Å) relative to the co-crystalline ligand. Depicting well-matching superimposition (Figure 6B), the above-cited ligand-residue hydrophobic and polar binding interactions were conserved for the redocked acarbose molecule following the molecular docking protocol (Table 1). Additionally, the polar functionalities of the redocked acarvosine and +2 maltose molecule illustrated close proximity (~4.7 Å) towards Asp203, Asp443, Asp571, and Tyr605 allowing a good chance of forming water-mediated hydrogen-bonding. Based on these redocking findings, the adopted docking protocol was confirmed appropriate since depicting RMSD values below 2.0 Å indicated that both the adopted docking parameters and algorithms were sufficient for determining the best docking pose [58]. Thus, the adopted directed docking protocol can ensure the biological relevance of the obtained docking binding modes and in turn their respective docking energies.

Moving towards the molecular docking analysis of 1,3,6-trigalloylglucose (**5**), a more favorable docking score (−7.3 kCal/mol) was assigned for this gallotannin derivative as compared to the co-crystalline hMGAM inhibitor, acarbose. This compound predicted significant anchoring at the −1 and +1 subsites of the target protein (Figure 6C). Having its 1-O-galloyl ring directed into the −1 subsite, the investigated gallotannin derivative exhibited an extended hydrogen bond network with several key pocket residues including the sidechains of Asp327, His600, and Tyr299, as well as the catalytic Asp542 being crucial for the enzyme’s acid/base catalysis (Table 1). On the other hand, the 3-O-galloyl ring predicted polar contact with the N-terminal β-sheet loop residue, Asp203, near the rim of the binding site. Whereas, the 6-O-galloyl moiety exhibited a single hydrogen bond pairing with the mainchain of Leu577 lining the +1 subsite. Both the catalytic residue, Asp542, and N-terminal β-sheet loop residue, Thr205, further stabilized the ligand’s central _D_-glucopyranose scaffold within the canonical pocket through strong hydrogen bond pairing with its C2 hydroxy group. Further stabilizing interactions were provided via comparable acarbose-based hydrophobic contact profiles with Tyr299, Ile328, Ile364, Trp406, Trp441, Phe450, Trp539, Phe575, Ala576, Leu577, and Tyr605. Nevertheless, only the docked gallotannin derivative predicted significant π-mediate hydrophobic interactions with Tyr299 and Phe575 owing to its aromatic nature.

Overlayed conformational analysis of the docked gallotannin derivative and co-crystalline acarbose showed a relevant orientation of the ligand’s 1-O-galloyl ring at the acarbose aglycone moiety relevant to the −1 subsite. Regarding the central _D_-glucopyranose scaffold of the docked gallotannin derivative, relevant orientation was depicted for this flexible core structure being at the intermediate distance of both the 6-deoxyglucosyl and first maltose moieties of the acarbose corresponding to the +1 and +2 carbohydrate subsites, respectively. Interestingly, both C3 and C6 galloyl rings showed no typical orientation at the +3 subsite, this was due to the extended and branched topology of the docked gallotannin compound allowing minimal steric clashes with the lining residues of the substrate-binding pocket. It is worth mentioning, that only a 3-O-galloyl ring was in close proximity to the latter subsite residues. Depicting such a favored docking pose, the docked gallotannin compound depicted a V-shaped/arrow-head conformation within the hMGAM binding site having its 1-O-galloyl ring as its anchor while both other galloyl rings were flanked at both sides of the compound. Accumulated evidence from the crystalline structures of GH-31 family homologs and lysosomal α-glucosidase enzymes revealed no direct interactions between co-crystallized inhibitors and +3 subsite residues while suggesting the paucity of only −1 and +1 subsites as the productive substrate-binding sites for these biological targets [51,53,54,55,56]. Thus, the lack of predicted direct polar contacts for the isolated gallotannin derivative with +3 carbohydrate subsite residues was suggested to not greatly influence the ligand-target affinity and neither its enzyme inhibition potentiality.

### 3.6. Molecular Dynamics Simulation Analysis

**Figure 7:**
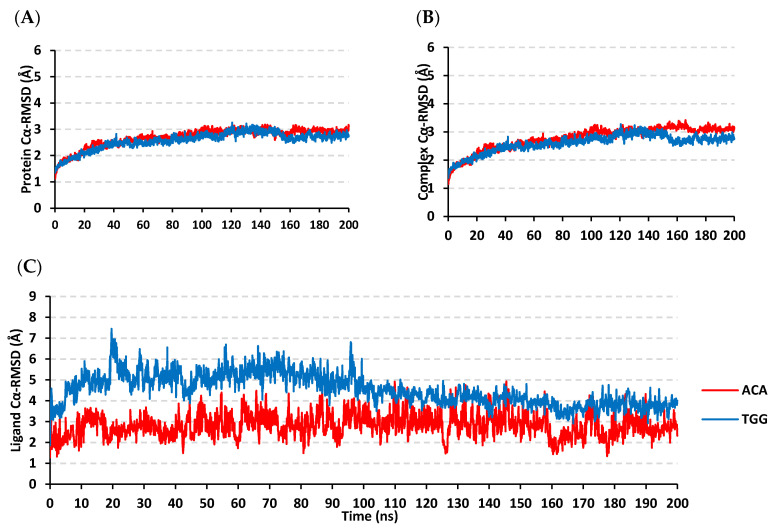
Stability analysis for the investigated gallotannin derivative and reference inhibitor, acarbose, in complex with *h*MGAM protein along 200 ns explicit MD simulation. (**A**) Protein *C*α-RMSDs; (**B**) Complex *C*α-RMSDs; (**C**) sole ligand *C*α-RMSDs (Å) in reference to protein backbone Cα-carbon atoms, across MD simulation time (ns); ACA (acarbose); TGG (1,3,6-trigalloyl glucose).

Both the docked gallotannin derivative and co-crystallized acarbose in complex with hMGAM were subjected to 200 ns molecular dynamics (MD) simulations. The estimated RMSD deviations for the target hMGAM proteins, in reference to the Cα backbone (Cα-RMSD), illustrated an overall typical behavior for MD simulations (Figure 7A). Following the release of constraining at the start of the MD production, the proteins’ Cα-RMSDs were elevated over initial frames and for the first 30 ns of the MD runs. Beyond that timeframe, steady protein Cα-RMSD tones were obtained for more than half the simulation runs (i.e., >140 ns) with no significant fluctuations along with the entire MD timeframes. Notably, the hMGAM proteins within both simulated models were leveled-off at comparable Cα-RMSDs throughout the trajectory plateau and till the MD simulation end (2.8 ± 0.1 Å, and 2.7 ± 0.2 Å for acarbose and gallotannin derivative models, respectively. These illustrated dynamic behaviors confer successful convergence of the hMGAM proteins throughout the designated MD simulation timeframes as well as successful system minimization, relaxation, and thermal equilibration prior to the MD production step. In these regards, no further extension of the MD simulation beyond the 200 ns-period was needed.

For investigating the stability of the docked gallotannin derivative and co-crystalline acarbose within the hMGAM substrate-binding site, the Cα-RMSDs of both the sole ligand as well as the combined ligand-hMGAM complex were monitored along with the whole MD timeframes in relation to the reference protein backbone frame (Figure 7B,C). Backbone Cα-RMSD plateaus were illustrated for both models, despite limited fluctuations, conferring significant confinement of the simulated ligands within the target’s pocket. Steady complex Cα-RMSDs tones were depicted for both ligands around averages of 2.7 ± 0.2 Å and 2.9 ± 0.2 Å for the investigated gallotannin derivative and acarbose, respectively. Comparable acarbose-based dynamic findings were also reported by Zhang et al. for unraveling possible inhibitors of the hMGAM biological target [59]. Concerning the sole ligand’s Cα-RMSDs, higher average values were assigned for the docked gallotannin compound in relation to the co-crystalline acarbose (5.2 ± 0.5 Å versus 2.9 ± 0.6 Å) till the half of the MD simulation run. However, the simulated gallotannin derivative depicted more steady and equilibrated trajectories beyond the 100 ns timeframe and till the end of the simulation as it managed to converge at lower Cα-RMSD around the final frames (3.8 ± 0.3 Å). On the other hand, ligand’s Cα-RMSDs fluctuations with higher standard deviation values were shown for the acarbose across the whole MD simulation run, highlighting possible conformational changes for the pseudosugar within the hMGAM substrate-binding pocket. The presented Cα-RMSD trajectories ensured successful convergence, as well as ligand-pocket confinement and the suitability of 200 ns MD simulation, runs without the need for further extensions.

**Figure 8:**
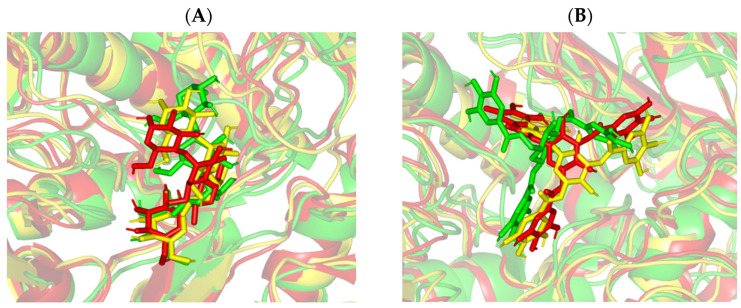
Conformational analysis of simulated ligand-hMGAM complexes at the initial, middle, and final snapshots. Overlaid snapshots of the ligand-protein complexes; (**A**) acarbose; (**B**) 1,3,6-trigalloylglucose, at 0 ns, 100 ns, and 200 ns MD simulation frames. The target proteins are represented in green, yellow, and red cartoons corresponding to the initial, midway, and last extracted frames, respectively. Ligands (sticks) are presented in colors corresponding to their respective extracted frames.

Examining the models at trajectories of the start, midway, and final timeframes (0 ns, 100 ns, and 200 ns), was beneficial for monitoring the possible conformational changes of the simulated ligand-protein models across the MD simulation run. Designated frames for each ligand-protein model were extracted and minimized to a 0.001 kcal/mol.A^2^ gradient using the MOE system preparation package. Notably, stable ligand-hMGAM binding profiles were assigned for both the simulated gallotannin derivative and co-crystalline ligand (Figure 8). Ligands showed favored orientation/conformation at the hMGAM substrate-binding pocket, while limited ligand orientation alterations were depicted at the end of the MD simulation runs. Throughout the entire MD simulation run, the co-crystalline ligand acarbose showed significant conformational alterations for its terminal +3 maltose sugar as compared to the rest of the ligand’s scaffold rings (Figure 8A). This dynamic behavior could be reasoned for the reported limited binding interaction at this terminal subsite which matches the above molecular docking investigation as well as the reported crystalline structures [51,53,54,55,56]. The reasonably flexible +3 maltose ring could account for the significant acarbose’s Cα-RMSDs fluctuations having higher standard deviation values across the MD simulation run. On the one hand, the ligand’s acarvosine scaffold (aglycone valienamine and 6-deoxyglucosyl moieties) was highly confined at the −1/+1 sugar subsites showing limited conformational/orientation shifts. Moving towards the simulated gallotannin derivative, the ligand’s V-shaped/arrow-head conformation was conserved showing the 1-O-galloyl moiety stably anchored at the −1 sugar subsite. On the other hand, both the central glucosyl scaffold and the other two flanking galloyl moieties showed reasonable conformational alterations yet maintained conserved orientations and confinements at the +1 and +2 sugar subsites.

**Figure 9:**
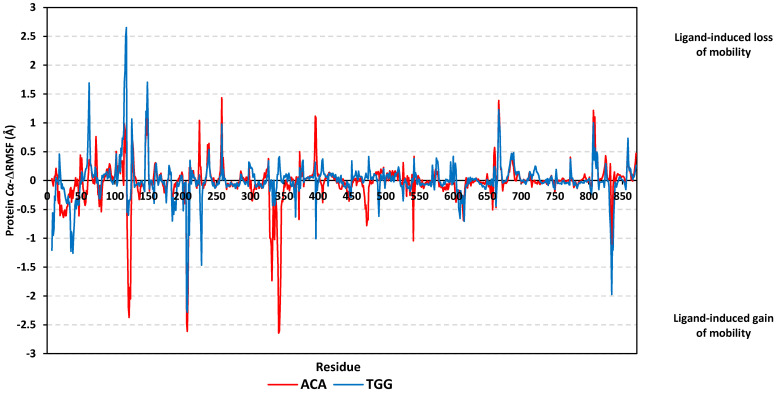
Protein ΔRMSF analysis, in complex with investigated gallotannin derivative and acarbose, across 200 ns MD simulation run. The ΔRMSF values, in reference to protein backbone Cα-atoms, were estimated and represented in terms of residue sequence numbers.

For further investigation of the hMGAM’s local flexibility and residue-wise contribution within the ligand-protein binding stability, the difference root-mean-square fluctuations (ΔRMSF) of the apo hMGAM relative to its holo state (RMSF_Apo_−_Holo_) were monitored along the whole MD runs. Applying the cut-off mobility threshold at ΔRMSF of 0.3 Å, higher flexibility/mobility patterns were assigned for the N-terminal residues as compared to those of the carboxy end (Figure 9). This was consistent with the MD dynamic behavior of the hMGAM proteins reported by Zhang and his research group [59] as well as the reported conformational stability and B-factor analyses regarding the hMGAM crystalline structures [51,53,54,55,56]. The latter would highlight the validity of the presented MD simulation study and adopted protocol. Regarding the core protein residues, comparable flexibility/immobility profiles were depicted for both gallotannin derivative and acarbose models across several residue ranges. Notably, the residue ranges along 200–210 and 830–850 showed significantly high mobility profiles for both ligands with ΔRMSF down to −2.6 Å. On the contrary, residues around 650–665 and 800–820 ranges were fewer mobiles (ΔRMSF up to 1.4 Å). On the other hand, trends of less negative and/or more positive ΔRMSF values were assigned for the amino acids in complex with gallotannin derivative relative to those of the simulated acarbose reference ligand. This was most recognized across 55–60 and 105–117 residue ranges where the gallotannin-bound protein residues exhibited highly positive (ΔRMSF ~2.7 Å versus 0.3 Å) or either much less negative ΔRMSF (~−0.4 Å versus −2.6 Å) across the 118–124, and 330–350 residue ranges. The latter findings further highlight the impact of significant acarbose fluctuations on the protein tertiary structure stability.

Notably, the 330–350 residue range originated from the ligand-binding domain (catalytic GH-31) was almost three-fold higher immobility profile assigned for the gallotannin-protein model in relation to that of acarbose. This differential fluctuation/immobility pattern infers the significant role of these residues for gallotannin binding at the hMGAM substrate-binding site. Focusing on the specific flexibility of the pocket’s key lining residues in relation to bounded ligand, interesting findings were also illustrated. The simulated gallotannin compound depicted a wider residue range of immobility/inflexibility as compared to acarbose (Table 2). The Pocket’s residues; Arg298, Asp327, Asp542, and His600 showed recognized inflexibility profiles (ΔRMSF ≥ 0.3 Å) for both ligands which highlight their crucial role in ligand anchoring at −1 sugar subsite. Only Arg5276 showed a significant immobility value at acarbose being higher than at the galloyannin model. On the other hand, residues like Thr211, Phe575, Ala576, Leu577, Gln603, Trp406, and Phe450 showed immobility values beyond the cut-off ΔRMSF values only for the gallotannin derivative model. Based on the hydrophobic/hydrophilic nature of the depicted immobilized residues, trends of polar residues were dominant for stabilizing the acarbose-protein model, while both polar and hydrophobic amino acids were shown important for gallotannin stability. Finally, the above residue-wise immobility profiles inferred the important role of catalytic GH-31 domain residues, with the assistance of particular residues of the N-terminal β-domain and both inserted loops, for stabilizing the simulated ligands at the hMGAM substrate-binding site.

To investigate the binding affinity of the simulated ligands towards hMGAM as well as grasp the nature of ligand interactions, the binding-free energy was estimated by MD-directed MM/PBSA calculations [60]. This approach can account for more accurate ligand-protein affinity as compared to static or highly sophisticated flexible docking techniques but is computationally less expensive than free energy perturbation [41]. The SASA-only model of the free-binding energy calculation (ΔG_Total_ = ΔG_Molecular Mechanics_ + ΔG_Polar_ + ΔG_Apolar_), and single trajectory approach, were adopted. Interestingly, a more favorable total binding affinity was estimated for the simulated gallotannin derivative as compared to the reference inhibitor (−69.6 ± 24.9 versus −40.7 ± 22.8 kJ/mol). The latter binding free energy pattern matches the trend from the preliminary docking investigation showing preferential higher docking scores for 1,3,6-trigalloylglucose in relation to acarbose.

Dissecting the obtained ligand-hMGAM binding-free energy into its contributing energy terms (Table 3) has shown a dominant energy contribution of the electrostatic interactions (ΔG_Electrostatic_) within the free-binding energy calculation for acarbose. This electrostatic energy term was more than five folds higher than that of the van der Waals (ΔG_Van der Waal_) contributions for the acarbose-protein model. On the other hand, the simulated gallotannin derivative showed balanced electrostatic/van der Waal energy term contributions. It is worth mentioning that these energy findings were consistent with the above described ΔRMSF hydrophobic/polar contact preferentiality. Higher polar solvation energy (ΔG_Solvation; Polar_) was assigned for acarbose, whereas the non-polar solvation energy (ΔG_Solvation; SASA_) was higher for the simulated gallotannin derivative. The high ΔG_Solvation; Polar_ depicted with acarbose can be reasoned since the ligand possesses several polar oxygen-related functionalities. While these groups can serve in furnishing polar contact with −1 and 1 sugar subsites, they can act as double blades increasing solvation entropy particularly since the hMGAM pocket is shallow and solvent-exposed. The latter could compromise the acarbose binding at the target site as binding is considered a solvent-displacement process. On the contrary, the simulated gallotannin derivative was assigned polar and hydrophobic functionalities of comparable magnitude (ΔG_Electrostatic_ ≈ ΔG_Van der Waal_). The ligand’s aromatic functionalities would provide reasonable hydrophobic compensation against the potential solvation entropy.

**Figure 10:**
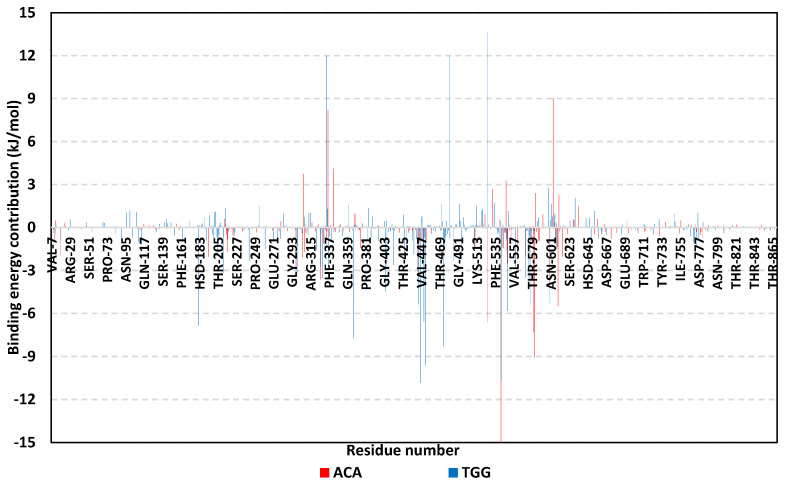
Residue−wise binding−free energy contributions for simulated ligand-*h*MGAM complexes.

For gaining more insights regarding ligand-residues interactions, the binding-free energy was further decomposed for identifying the key residue-wise binding energy contributions (Figure 10) [41]. Depicting high binding energy contribution (≥−3.0 kJ/mol), pocket’s residues including Tyr299, Asp327, Asp443, Asp542, Asp571, and His600, were considered significant for both ligand-protein complex stability. However, the energy contribution of several hydrophobic pocket residues; Thr211, Tyr299, Trp406, Met444, Phe450, Leu473, Phe575, and Leu577, were only significant for the simulated gallotannin derivative (up to −8.3 kJ/mol). The highest residue-wise energy contribution was assigned for the catalytic Asp542 pocket residue (up to −15.0 kJ/mol and −10.6 kJ/mol for acarbose and gallotannin compounds, respectively). Nevertheless, a few residues like; Arg334, Lys480, and Arg562 in the gallotannin model and Asp334 and Gly604 within the acarbose model, showed sizeable positive energy contribution. The latter infers a repulsion effect and an unfavored impact on the stability of respective ligand-target complexes.

## 4. Conclusions

Five major phenolic constituents: apeginin-7-O-β-D-glucoside (**1**), quercetin-3-O-β-D-glucuronic acid (**2**), quercetin-3-O-β-D-galacturonic acid (**3**), rutin (**4**), and 1,3,6-trigalloyl glucose (**5**) were purified and characterized from *C. oblongifolia*. Their antioxidant and AGEs formation, α-glucosidase, and lipase inhibitory capacities were estimated. Compound **5** possessed the highest antioxidant and AGEs inhibitory potential. The furnished molecular docking and dynamics simulations illustrated the preferential stability and binding affinity of compound **5** towards human α-glucosidase in relation to its reported potent inhibitor, acarbose. The latter findings suggest the potential therapeutic utility of the *C. oblongifolia*-isolated compound for managing diabetes mellitus as well as other obesity-related conditions.

## Figures and Tables

**Figure 1:**
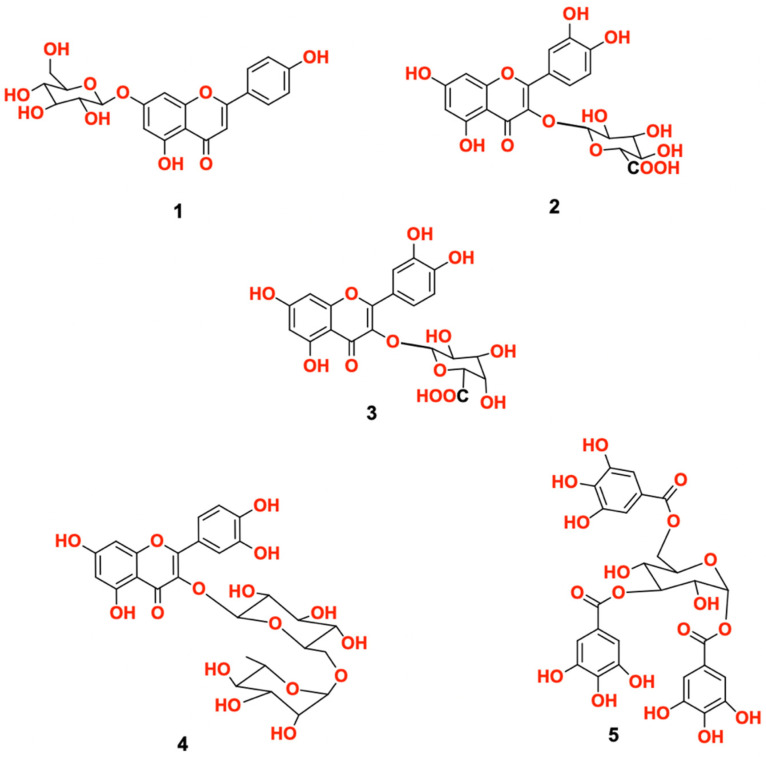
Isolated compounds **1**–**5** from *C. oblongifolia*.

**Table 1:** Docking energies and descriptions of ligand-hMGAM binding interactions.

Ligand	Docking Energy (Kcal/mol)	H-Bond Interactions [Length (Å); Angle (°); Binding Residues]	Hydrophobic Interactions	π-Interactions
Acarbose	−6.8	1.9 Å; 159°; Asp203 (sidechain CO^−^/6-deoxyglucosyl 3′-OH) 2.0 Å; 160°; Asp203 (sidechain CO^−^/6-deoxyglucosyl 4′-OH) 2.1 Å; 164°; Thr205 (sidechain OH/+3 maltosyl 6′-OH) 1.9 Å; 175°; Asp327 (sidechain CO^−^/valienamine 4′-OH) 2.0 Å; 168°; Arg526 (sidechain =NHH/6-deoxyglucosyl 3′-OH) 2.0 Å; 146°; Arg526 (sidechain =NHH/valienamine 6′-OH) 1.9 Å; 142°; Asp542 (sidechain CO^−^/glycosidic linker NH) 1.7 Å; 155°; Asp542 (sidechain C=O/valienamine 6′-OH) 2.3 Å; 142°; His600 (sidechain NH/valienamine 4′-OH) 2.3 Å; 138°; His600 (sidechain NH/valienamine 5′-OH)	Tyr299, Ile328, Ile364, Trp406, Trp441, Phe450, Trp539, Phe575, Ala576, Leu577, Tyr605	-
1,3,6-Trigalloyl glucose	−7.3	2.5 Å; 145°; Asp203 (sidechain CO^−^/C3-galloyl 3′-OH) 3.2 Å; 124°; Thr205 (sidechain OH^−^/sugar C2-OH) 2.5 Å; 154°; Tyr299 (sidechain OH/C1-galloyl 6′-OH) 2.3 Å; 127°; Asp327 (sidechain CO ^−^/C1-galloyl 4′-OH) 2.5 Å; 154°; Asp542 (sidechain CO ^−^/sugar C2-OH) 2.5 Å; 137°; Asp542 (sidechain C=O/C1-galloyl 3′-OH) 2.0 Å; 157°; Leu577 (mainchain NH/C6-galloyl 3′-OH) 3.2 Å; 144°; His600 (sidechain NH/C1-galloyl 4′-OH)	Tyr299, Ile328, Ile364, Trp406, Trp441, Phe450, Trp539, Phe575, Ala576, Leu577, Tyr605	Tyr299 (H-π) Phe575 (H-π)

**Table 2:** Estimated ΔRMSF ^a^ values [Å] for ligand-hMGAM proteins along 200 ns MD simulation.

Canonical Domains Forming Substrate-Binding Site	Comprising Residues	Acarbose	1,3,6- Trigalloylglucose
**N-terminal *β*-sheet domain**	Arg202	−0.3	−0.1
	Asp203	−0.4	−0.0
	Thr204	−0.1	−0.1
	Thr205	−0.0	−0.0
	Pro206	−2.5	−2.2
	Asn207	−0.3	−0.1
	Asn209	−0.6	−0.1
	Thr211	0.2	**0.4**
	Tyr214	0.1	0.2
**Catalytic GH-31 domain**	Arg298	**0.3**	**0.3**
	Tyr299	0.0	**0.3**
	Asp327	**0.4**	**0.3**
	Ile328	−0.2	0.0
	Ile364	−0.0	−0.0
	Trp441	−0.1	−0.1
	Asp443	−0.3	−0.0
	Met444	−0.2	−0.0
	Ser448	−0.2	−0.1
	Arg526	**0.3**	−0.4
	Trp539	0.1	0.0
	Gly541	−1.0	0.0
	Asp542	**0.4**	**0.4**
	Asp571	0.0	−0.1
	Phe575	0.1	**0.4**
	Ala576	0.1	**0.3**
	Leu577	0.2	**0.3**
	Arg598	−0.0	−0.1
	His600	**0.3**	**0.4**
	Gly602	−0.1	0.2
	Gln603	−0.3	**0.3**
	Phe605	−0.3	−0.2
**Catalytic insert-I loop**	Val405	−0.1	0.2
	Trp406	−0.1	**0.4**
**Catalytic insert-II loop**	Ser448	−0.2	−0.1
	Phe450	−0.1	**0.3**
	Leu473	−0.7	0.7
	Asp474	−0.7	0.3

^a^ Relative difference root-mean-square fluctuation (ΔRMSF) was estimated for each ligand-associated hMGAM protein relative to the apo/unliganded state. Residues showing significant immobility are with ΔRMSF ≥ 0.30 Å cut-off are in bold and highlighted.

**Table 3:** Total binding-free energies and individual energy term of investigated gallotannin derivative and reference ligand at hMGAM substrate-binding site.

Energy (kJ/mol ± SD)	Ligand-Protein Complex	
	Acarbose	1,3,6-trigalloylglucose
**ΔG_van der Waals_**	−52.6 ± 10.9	−147.6 ± 20.2
**ΔG_Electrostatic_**	−280.1 ± 32.5	−165.2 ± 38.7
**ΔG_Solvation; Polar_**	311.6 ± 29.7	267.6 ± 56.3
**ΔG_Solvation; non-polar; SASA_**	−19.5 ± 0.8	−24.5 ± 3.5
**ΔG_Total binding_**	−40.7 ± 22.8	−69.6 ± 24.9

## Data Availability

Not applicable.

## Supplementary Materials

The following supporting information can be downloaded at: https://www.mdpi.com/article/10.3390/biology11050762/s1, Figure S1. Pancreatic lipase inhibitory effect of Orlistat; Figure S2. Concentration-response linear response of Ascorbic acid in ABTS assay; Figure S3. Concentration-response linear response of Ascorbic acid in FRAP assay; Figure S4. Concentration-response linear response of EDTA in metal chelation assay; Figure S5. Concentration-response linear response of Trolox in ORAC Assay; Figure S6. ^1^HNMR of compound 1 (DMSO, 850 MHz); Figure S7. ^13^C NMR of compound 1 (DMSO, 215 MHz); Figure S8. ^1^HNMR of compound 2 (DMSO, 850 MHz); Figure S9. ^13^C NMR of compound 2 (DMSO, 215 MHz); Figure S10. HSQC of compound 2 (DMSO, 850 MHz); Figure S11. HMBC of compound 2 (DMSO, 850 MHz); Figure S12. ^1^HNMR of compound 3 (DMSO, 850 MHz); Figure S13. ^13^C NMR of compound 3 (DMSO, 215 MHz); Figure S14. HSQC of compound 3 (DMSO, 850 MHz); Figure S15. HMBC of compound 3 (DMSO, 850 MHz); Figure S16. ^1^HNMR of compound 4 (DMSO, 850 MHz); Figure S17. ^13^C NMR of compound 4 (DMSO, 215 MHz); Figure S18. HSQC of compound 4 (DMSO, 850 MHz); Figure S19. HMBC of compound 4 (DMSO, 850 MHz); Figure S20. ^1^HNMR of compound 5 (DMSO, 850 MHz); Figure S21. ^13^C NMR of compound 5 (DMSO, 215 MHz); Figure S22. HSQC of compound 5 (DMSO, 850 MHz); Figure S23. HMBC of compound 5 (DMSO, 850 MHz), Table S1: Pancreatic Lipase inhibition assay by isolated compounds from *C. oblongifolia* and Orlistat; Table S2: Antioxidant activity of isolated compounds.
