# Supplementary material for: Phenolics from Chrozophora oblongifolia Aerial Parts as Inhibitors of α-Glucosidases and Advanced Glycation End Products: In-Vitro Assessment, Molecular Docking and Dynamics Studies
Source: Biology (Basel). 2022 May 17;11(5):762. doi: 10.3390/biology11050762 (PMC9139161; doi:10.3390/biology11050762)
Supplement: Supplementary file 1 [file biology-11-00762-s001.zip › biology-1676716-supplementary.pdf]

Article

Phenolics from *Chrozophora oblongifolia* Aerial Parts as Inhibitors of α-Glucosidases and Advanced Glycation End Products: *In-vitro* Assessment, Molecular Docking and Dynamics Studies

Albraa T. Kashegari[1], Akram A. Shalabi[2], Ali M. El-Halawany[2], Khaled M. Darwish[3], Mardi M. Algandaby[4], Sabrin R.M. Ibrahim[5,6], Gamal A Mohamed[1], Ashraf B Abdel-Naim [7], Sameh S. Elhady[1], Abdulrahman E. Koshak[1], Peter Proksch[8] and Hossam M. Abdallah[1,*]

[1] Department of Natural Products, Faculty of Pharmacy, King Abdulaziz University, Jeddah 21589, Saudi Arabia; gahussein@kau.edu.sa (G.A.M.); aekoshak@kau.edu.sa (A.E.K.); ssahmed@kau.edu.sa (S.S.E.)

[2] Department of Pharmacognosy, Faculty of Pharmacy, Cairo University, Giza 11562, Egypt; ali.elhalawany@pharma.cu.edu.eg; akramali2010@yahoo.com

[3] Department of Medicinal Chemistry, Faculty of Pharmacy, Suez Canal University, Ismailia 41522, Egypt; khaled_darwish@pharm.suez.edu.eg

[4] Department of Biological Sciences, Faculty of Science, King Abdulaziz University, Jeddah 21589, Saudi Arabia; malgandaby@kau.edu.sa

[5] Preparatory Year Program, Department of Chemistry, Batterjee Medical College, Jeddah 21442, Saudi Arabia; sabrin.ibrahim@bmc.edu.sa

[6] Department of Pharmacognosy, Faculty of Pharmacy, Assiut University, Assiut 71526, Egypt

[7] Department of Pharmacology and Toxicology, King Abdulaziz University, Jeddah 21589, Saudi Arabia; aaabdulalrahman1@kau.edu.sa

[8] Institute of Pharmaceutical Biology and Biotechnology, Heinrich-Heine-Universität Düsseldorf, Universitätsstrasse 1, 40225, Düsseldorf, Germany; Peter.Proksch@uni-duesseldorf.de

* Correspondence: hmafifi@kau.edu.sa

Supplementary data

Sample	% Inhibition<br>(50 μM)	IC50 (μM)<br>(Mean ± SE)
**1**	4.48	More than 50
**2**	1.92	More than 50
**3**	2.26	More than 50
**4**	NA	More than 50
**5**	NA	More than 50
**Orlistat**		0.70 ± 1.10

Table S1: Pancreatic Lipase inhibition assay of isolated compounds from *C. oblongifolia* and orlistat.

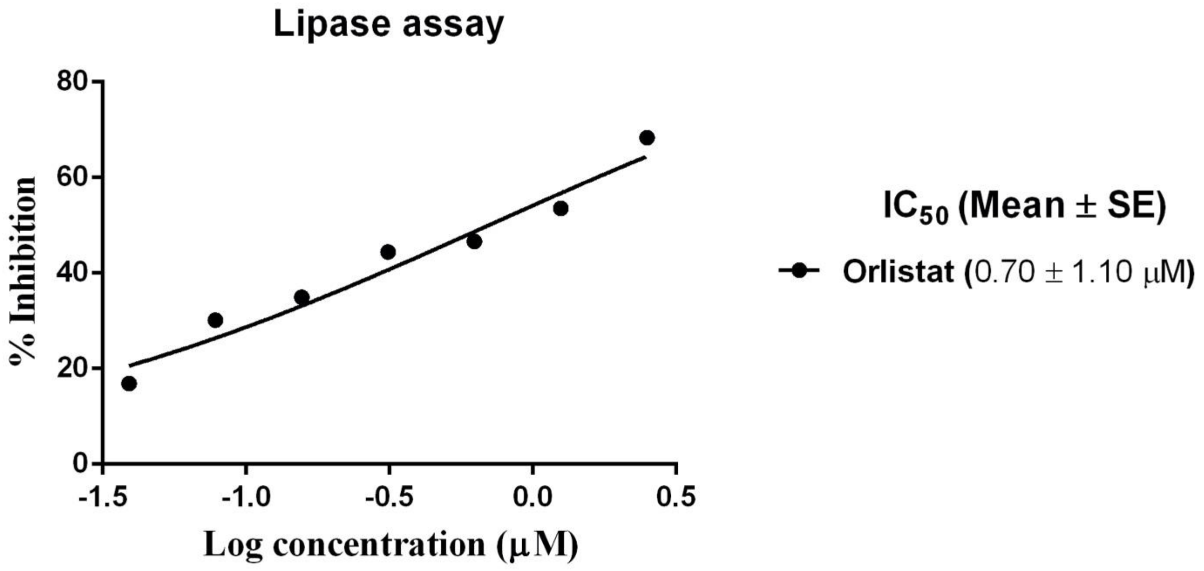

Figure S1. Pancreatic lipase inhibitory effect of orlistat.

Table S2: Antioxidant activity of isolated compounds (**1**-**5**).

**Compound**	**ABTS (mM AAE/mg)**	**FRAP (mM AAE/mg)**	**ORAC assay (*m*M TE/mg)**	**Metal chelation (µM EDTA eq/mg)**
**1**	0.702±0.050	0.225±0.003	1.092±0.067	**19.26±1.52**
**2**	0.646 ±0.072	0.727±0.021	0.739±0.098	**8.29±0.05**
**3**	0.210 ±0.012	0.494±0.066	0.538±0.015	**28.26±0.78**
**4**	1.972±0.059	2.658±0.168	2.164±0.096	**28.96±2.07**
**5**	9.640±0.061	7.228±0.279	1.119±0.116	**17.52±2.43**

AAE; Ascorbic acid equivalent; TE: Trolox equivalent

Concentration (µM)	% inhibition
7.8125	2.8930
15.625	3.7803
31.25	5.5156
62.5	9.7291
125	16.88018
250	33.5451
500	62.85834

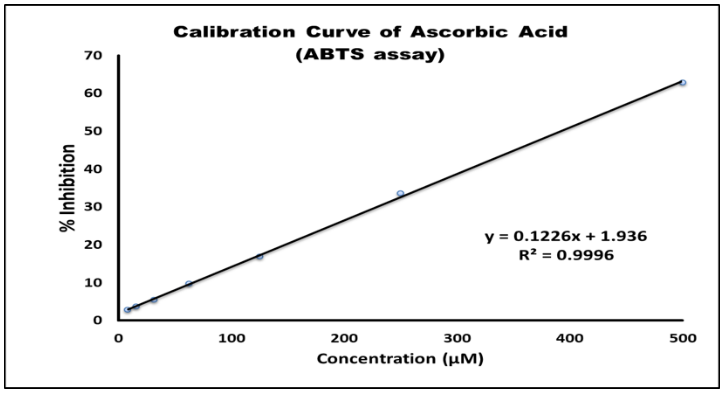

Figure S2. Concentration-response linear curve of ascorbic acid in ABTS assay.

Concentration (µM)	Absorbance
15.625	0.007
31.25	0.025
62.5	0.050
125	0.114
250	0.227
500	0.448

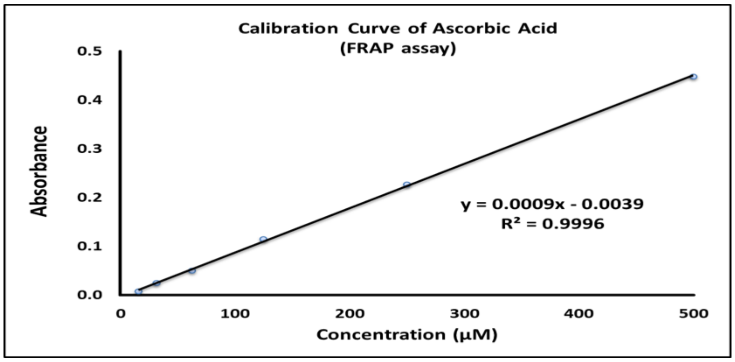

Figure S3. Concentration-response linear curve of ascorbic acid in FRAP assay.

Concentration (µM)	% inhibition
5	8.429
10	13.464
20	19.624
40	41.866
50	48.303
80	72.307

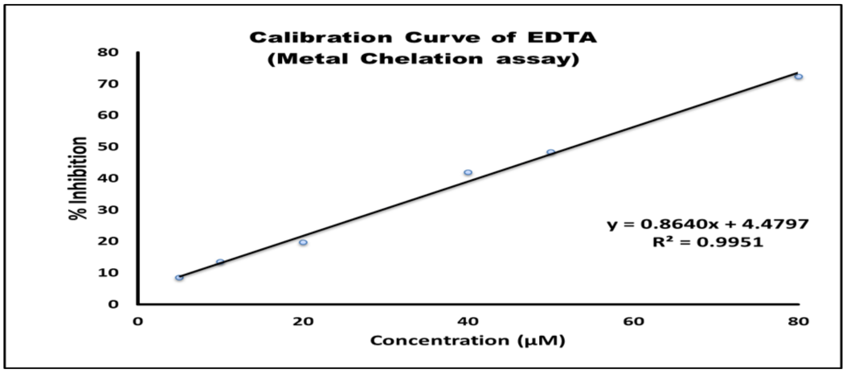

Figure S4. Concentration-response linear curve of EDTA in metal chelation assay.

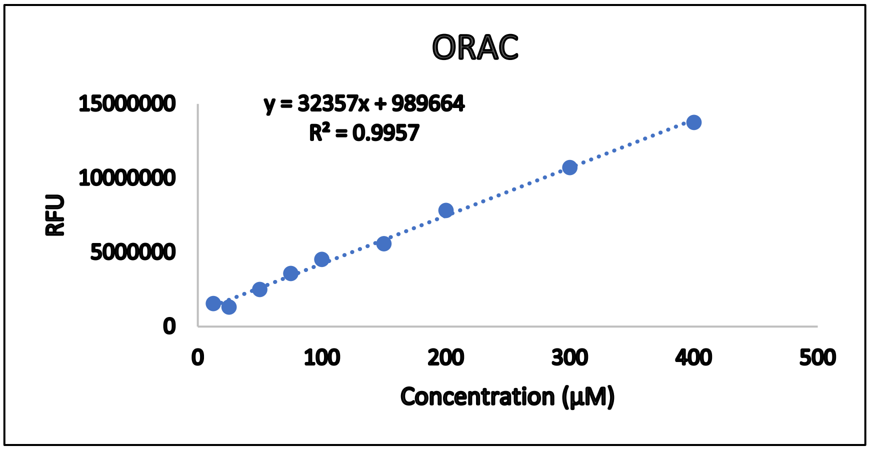

Figure S5. Concentration -response linear curve of trolox in ORAC assay.

Concentration (µM)	RFU
400	13762000
300	10720000
200	7830000
150	5576000
100	4530000
75	3580000
50	2507000
25	1314000
12.5	1557000

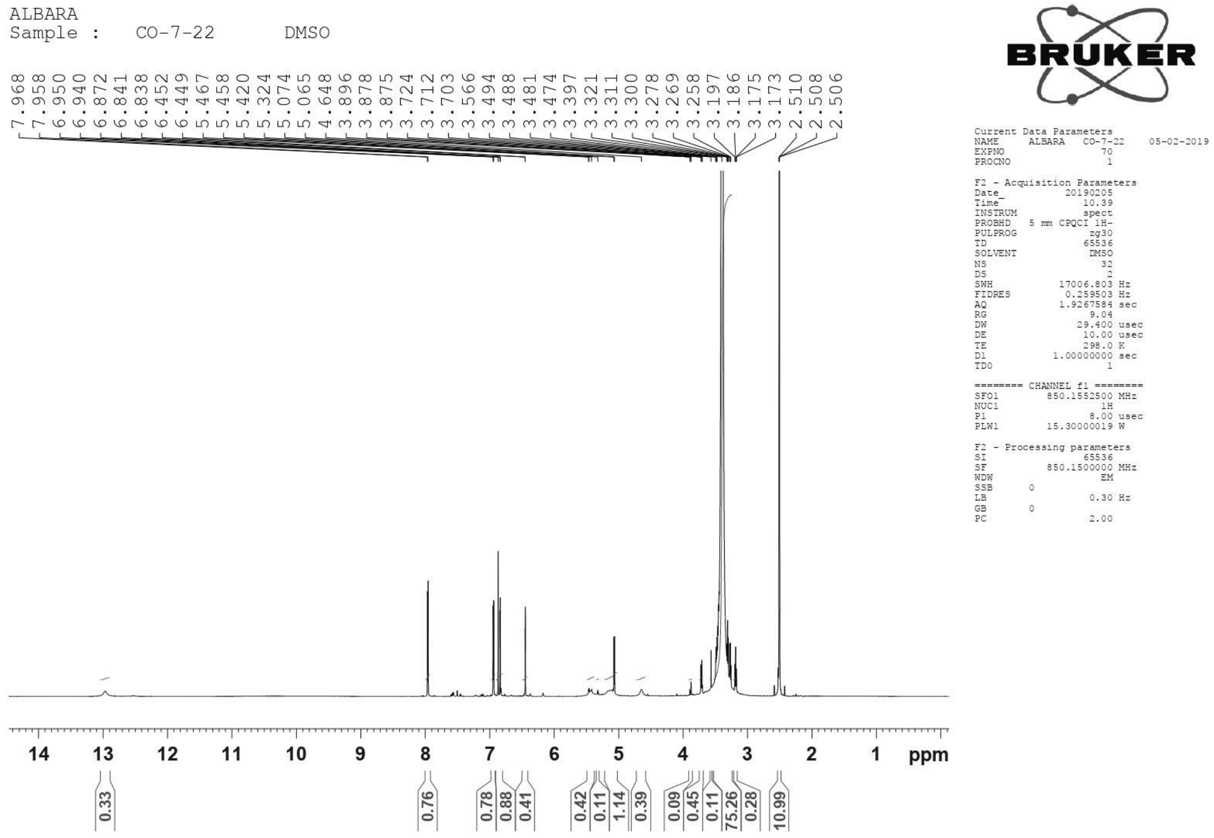

Figure S6. ^{1}HNMR of compound **1** (DMSO, 850 MHz).

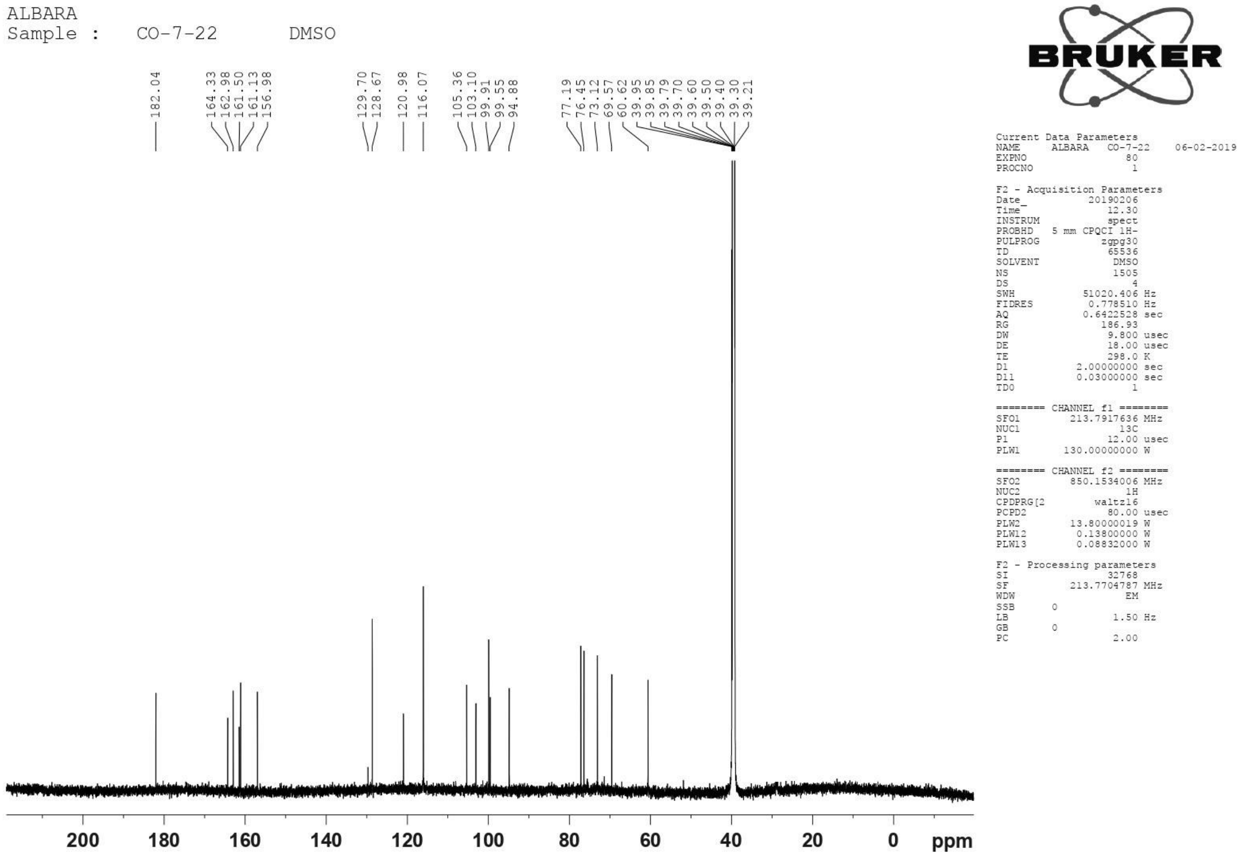

Figure S7. ^{13}C NMR of compound **1** (DMSO, 213 MHz).

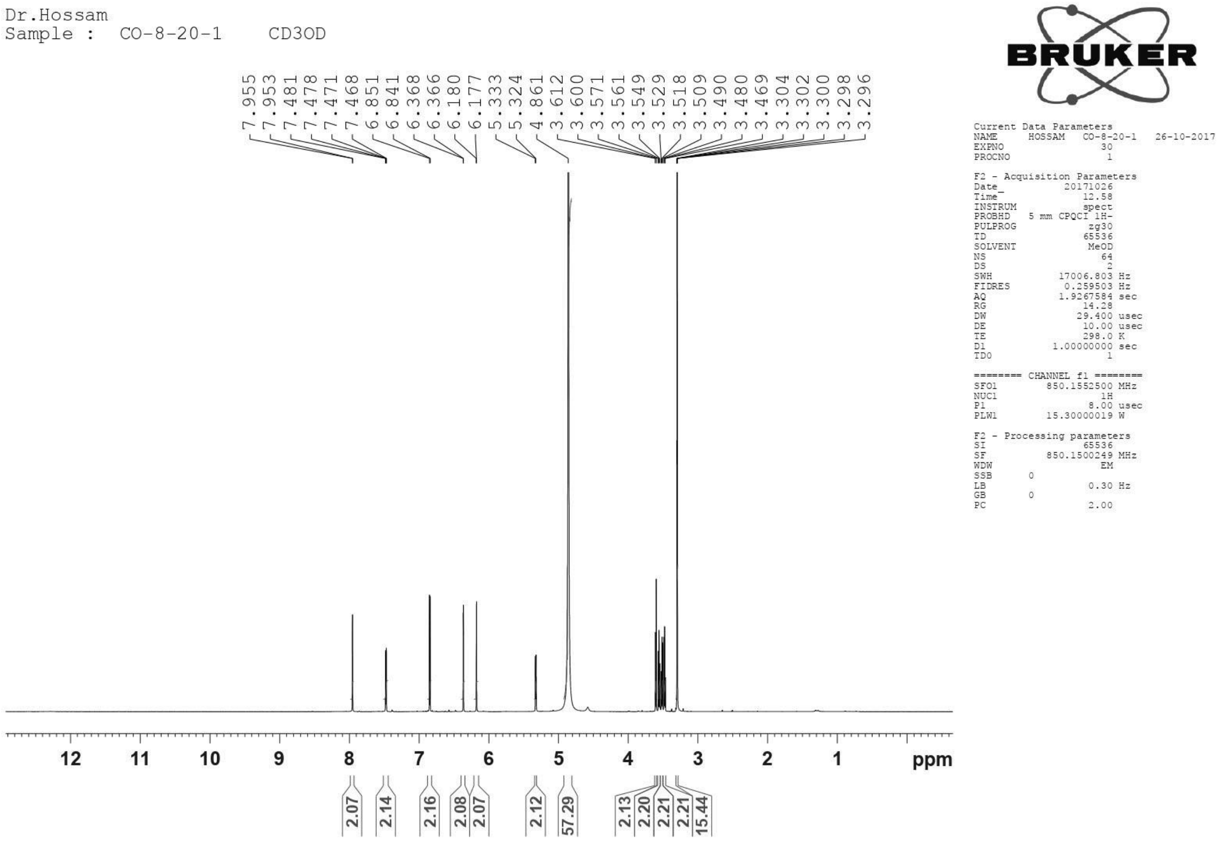

Figure S8. ^{1}HNMR of compound **2** (DMSO, 850 MHz).

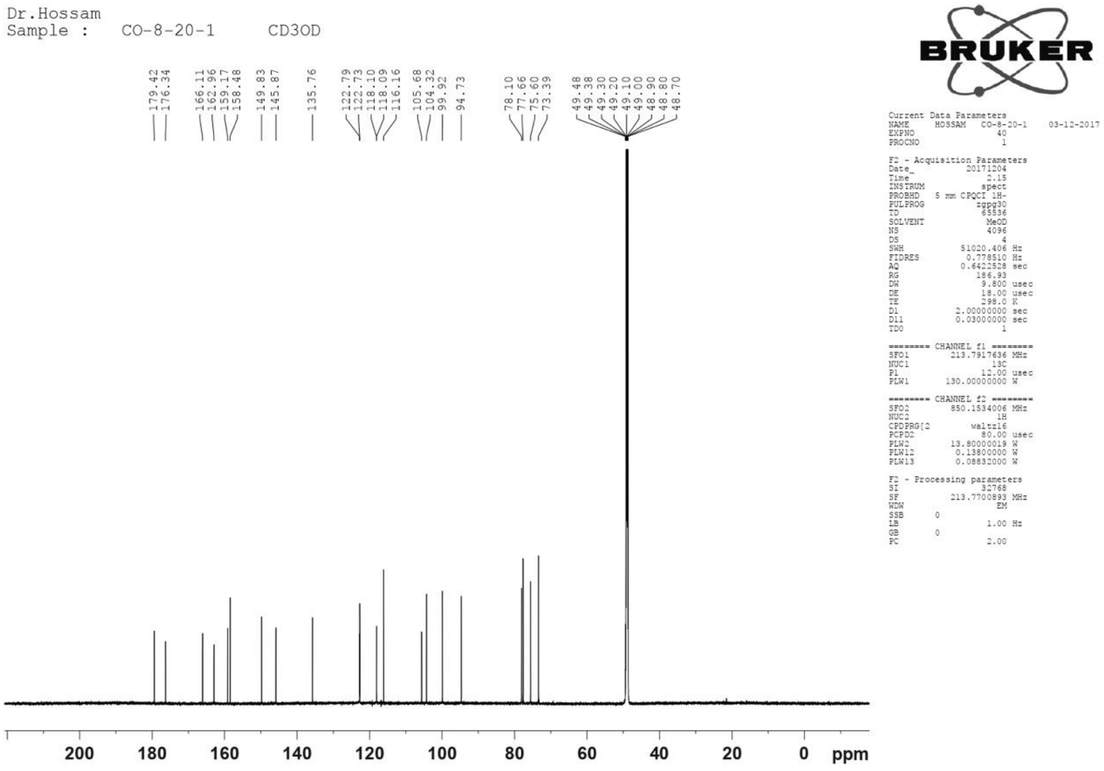

Figure S9. ^{13}C NMR of compound **2** (DMSO, 213 MHz).

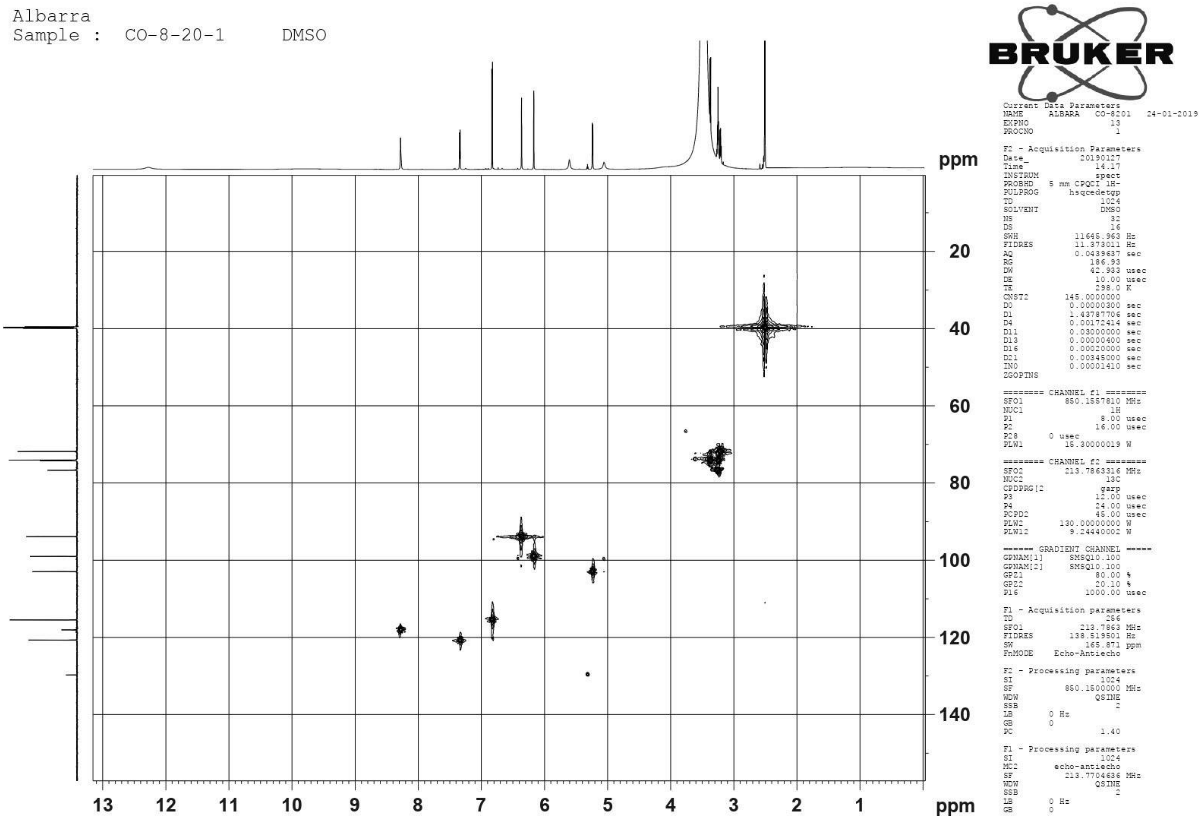

Figure S10. HSQC of compound **2** (DMSO, 850 and 213 MHz).

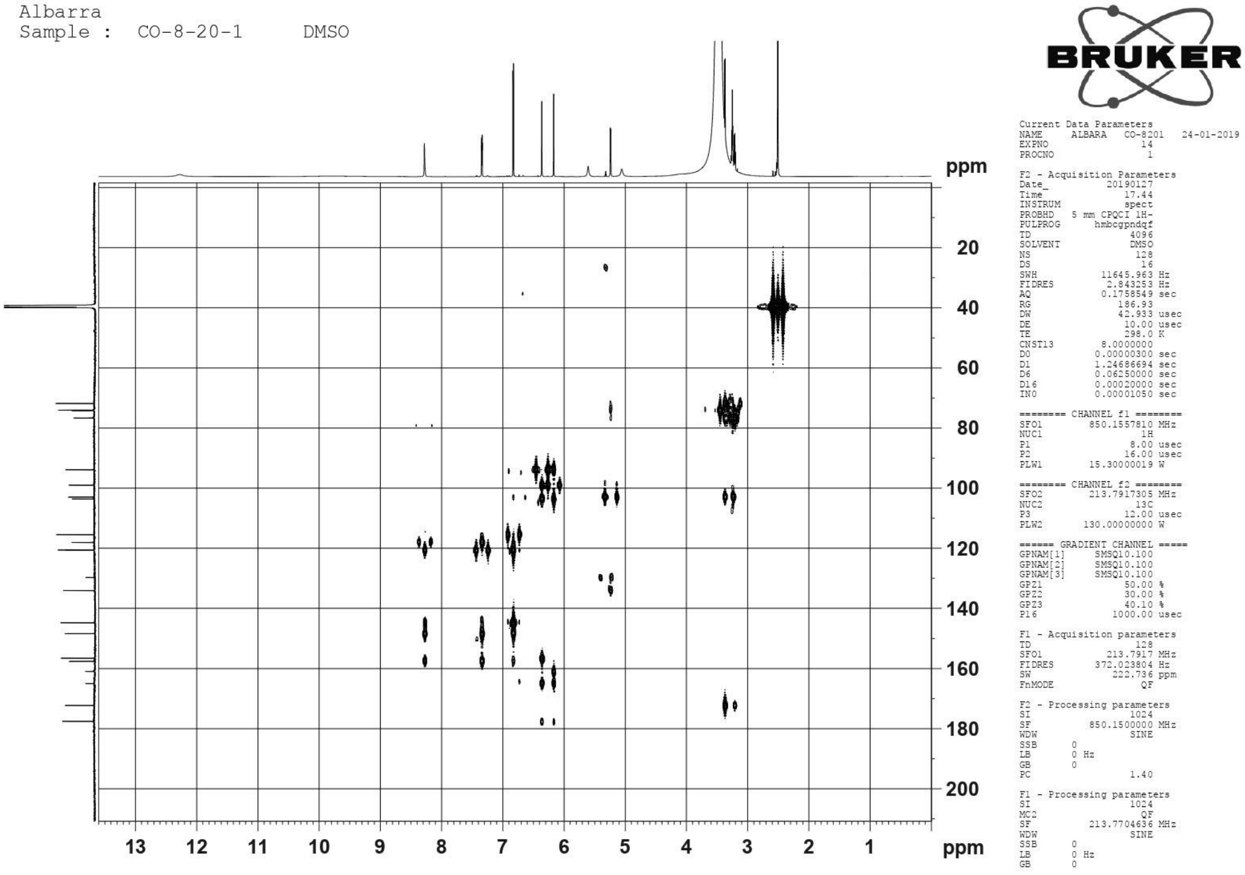

Figure S11. HMBC of compound **2** (DMSO, 850 and 213 MHz)

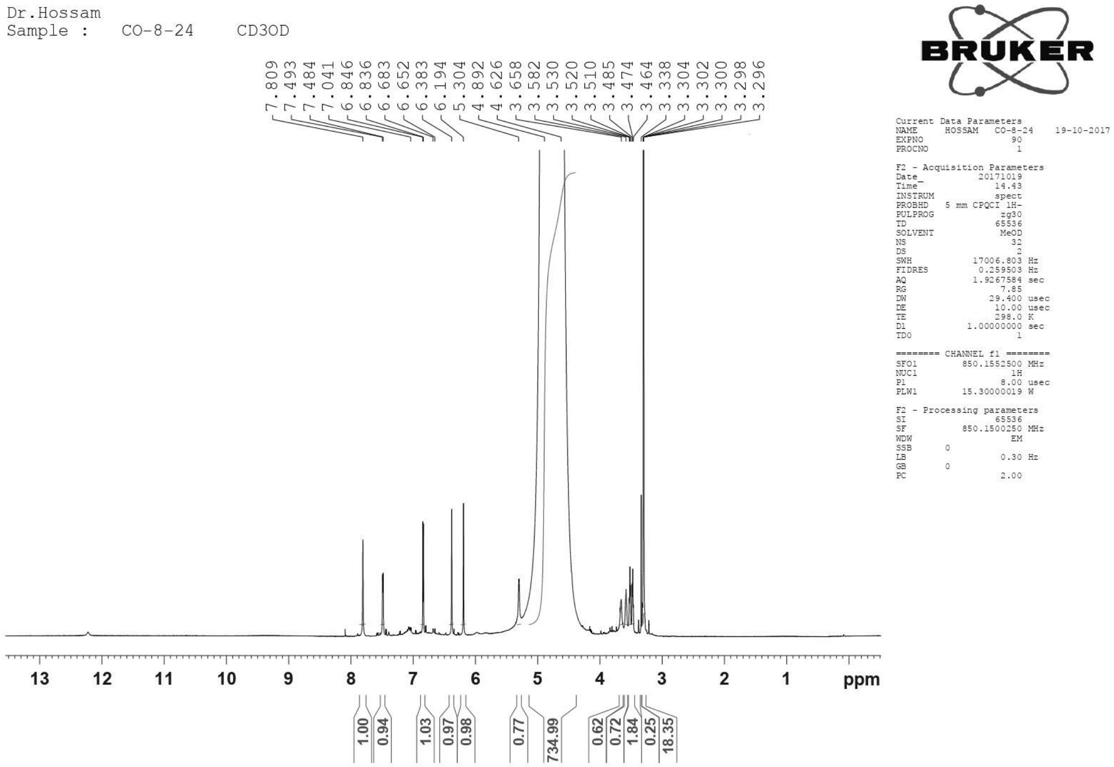

Figure S12. ^{1}HNMR of compound **3** (DMSO, 850 MHz)

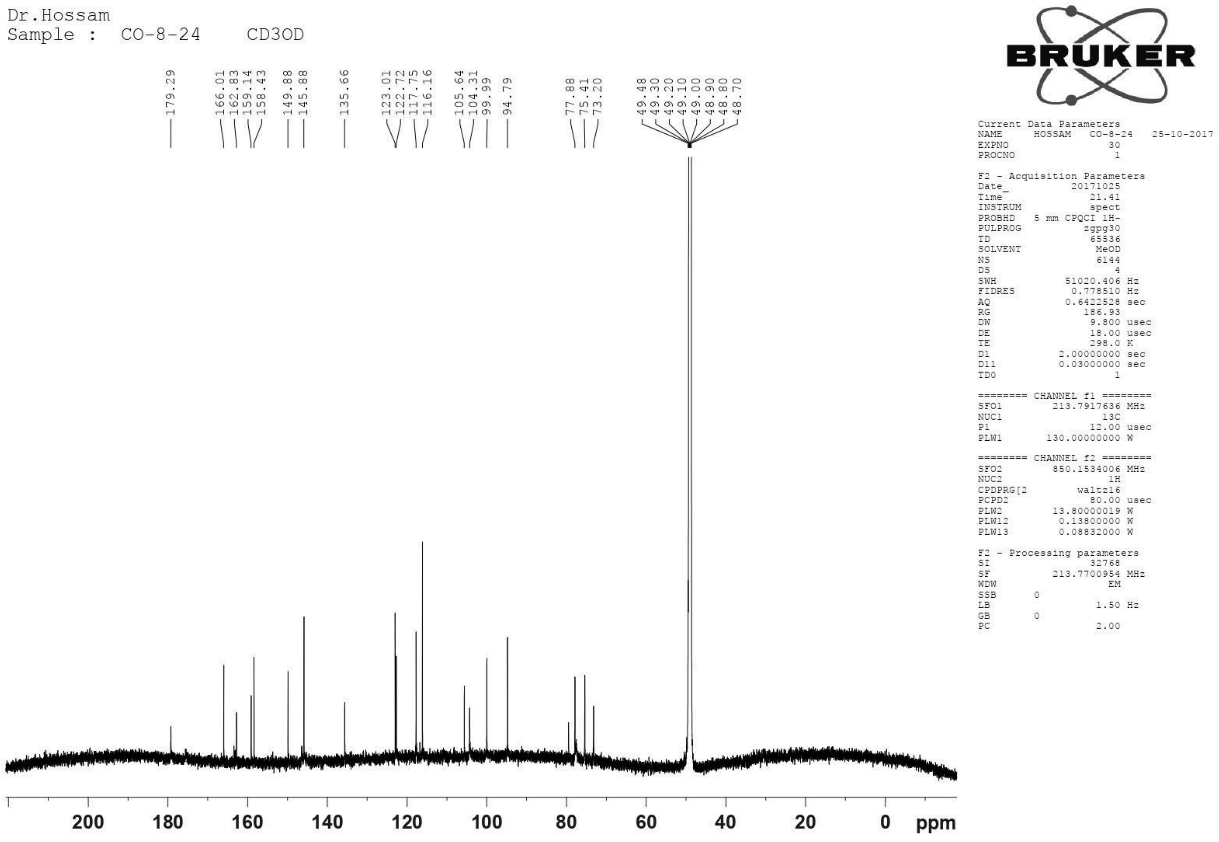

Figure S13. ^{13}C NMR of compound **3** (DMSO, 213 MHz)

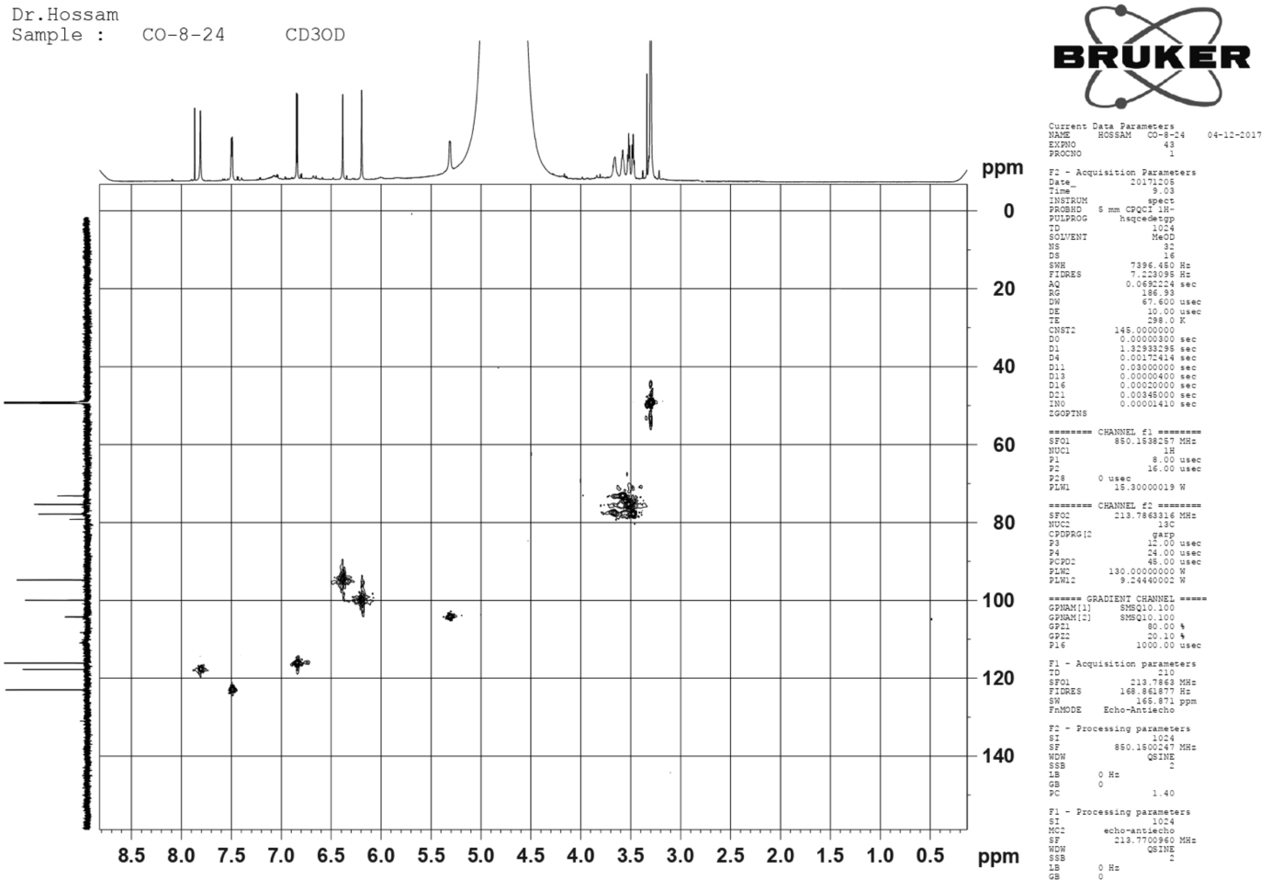

Figure S14. HSQC of compound **3** (DMSO, 850 and 213 MHz)

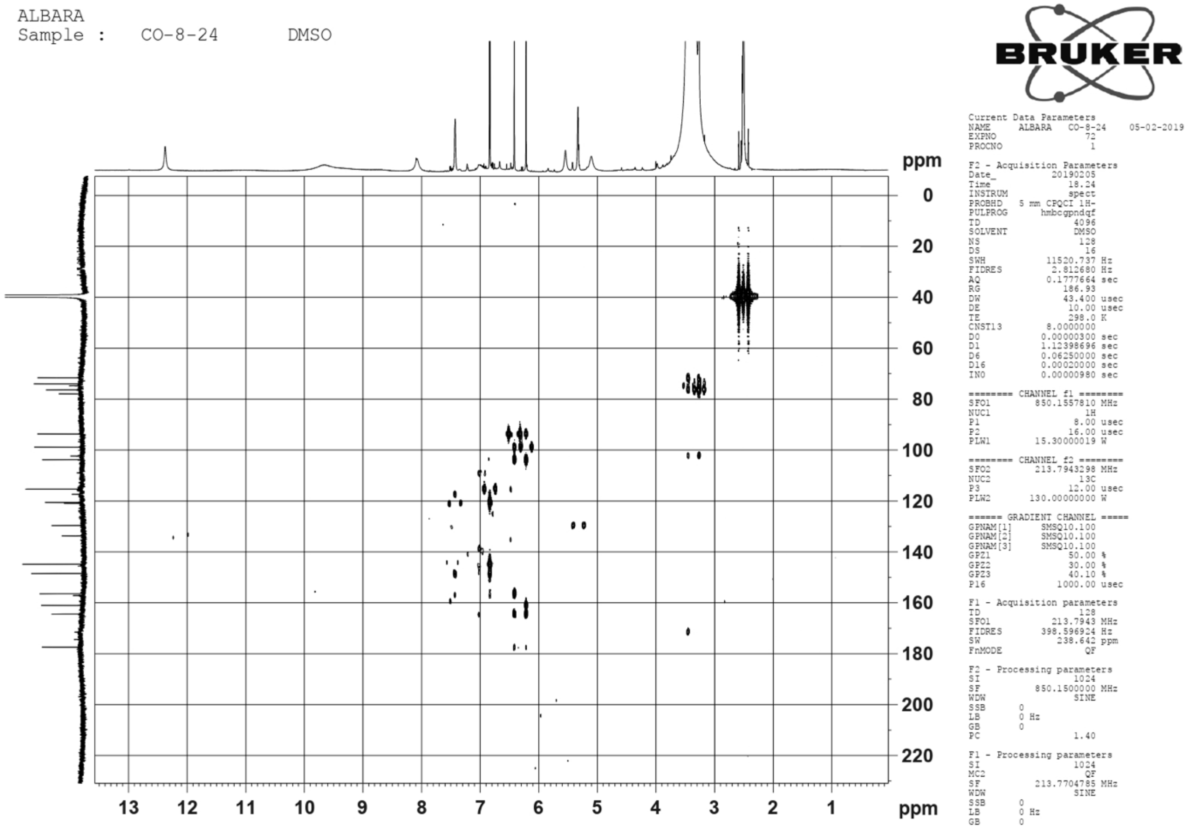

Figure S15. HMBC of compound **3** (DMSO, 850 and 213 MHz)

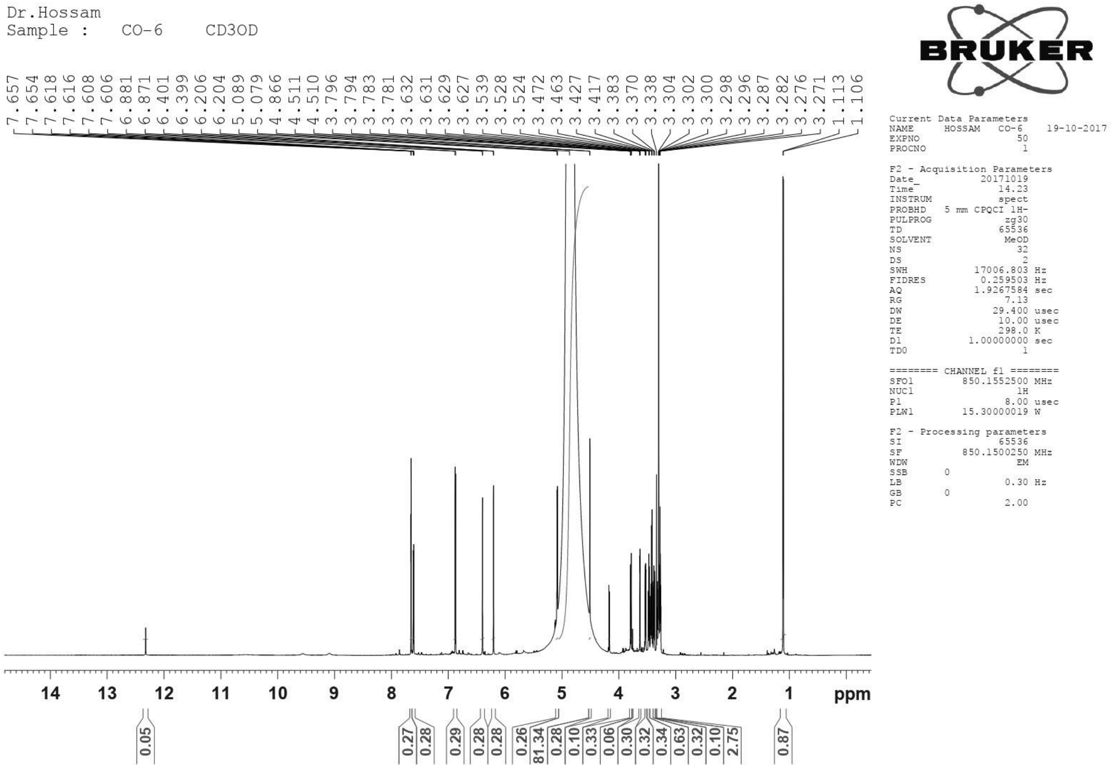

Figure S16. ^{1}HNMR of compound **4** (DMSO, 850 MHz)

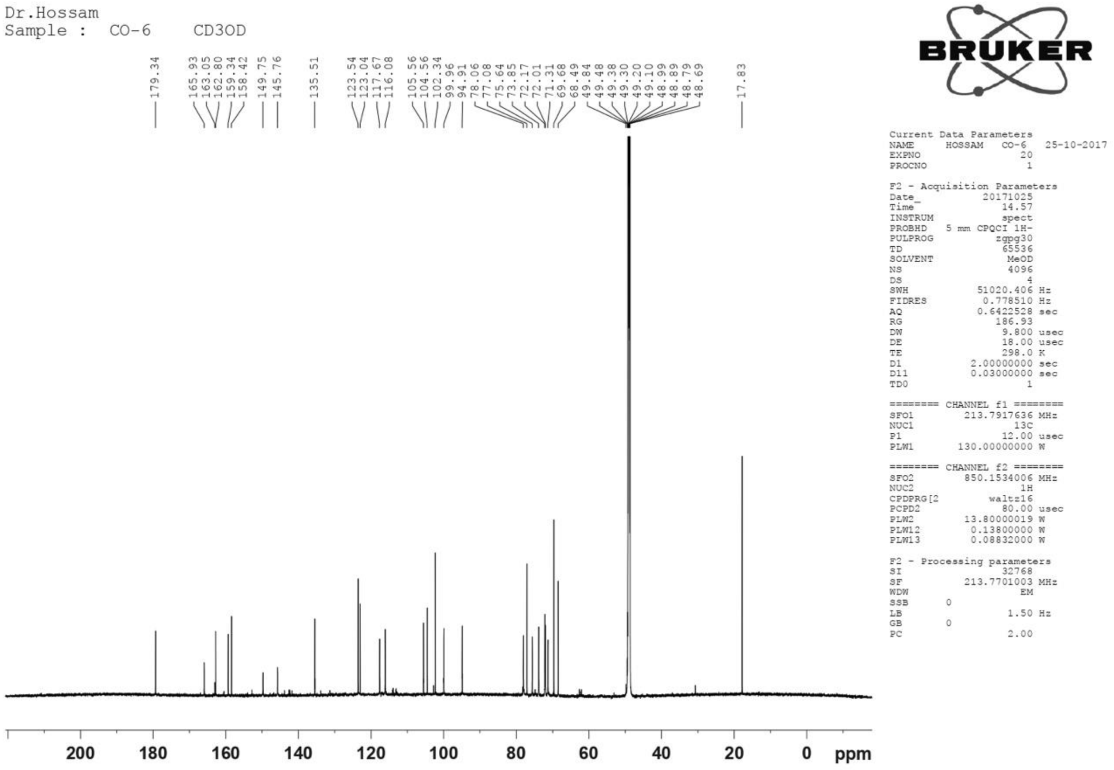

Figure S17. ^{13}C NMR of compound **4** (DMSO, 213 MHz)

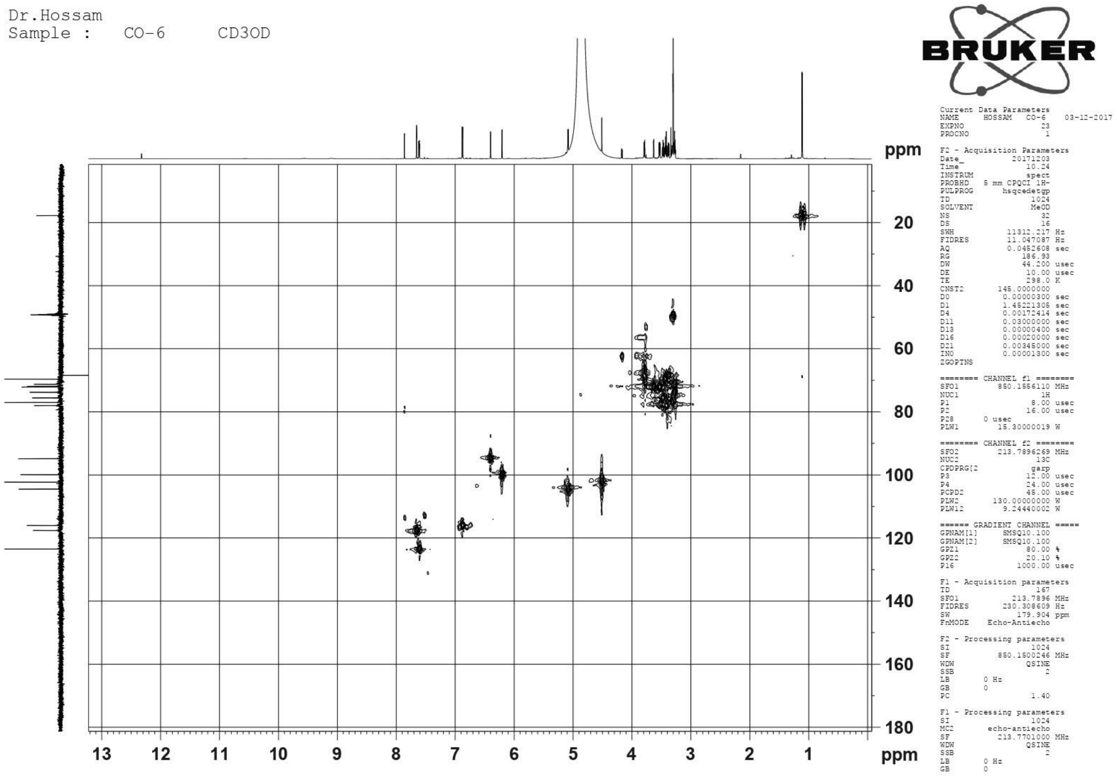

Figure S18. HSQC of compound **4** (DMSO, 850 MHz).

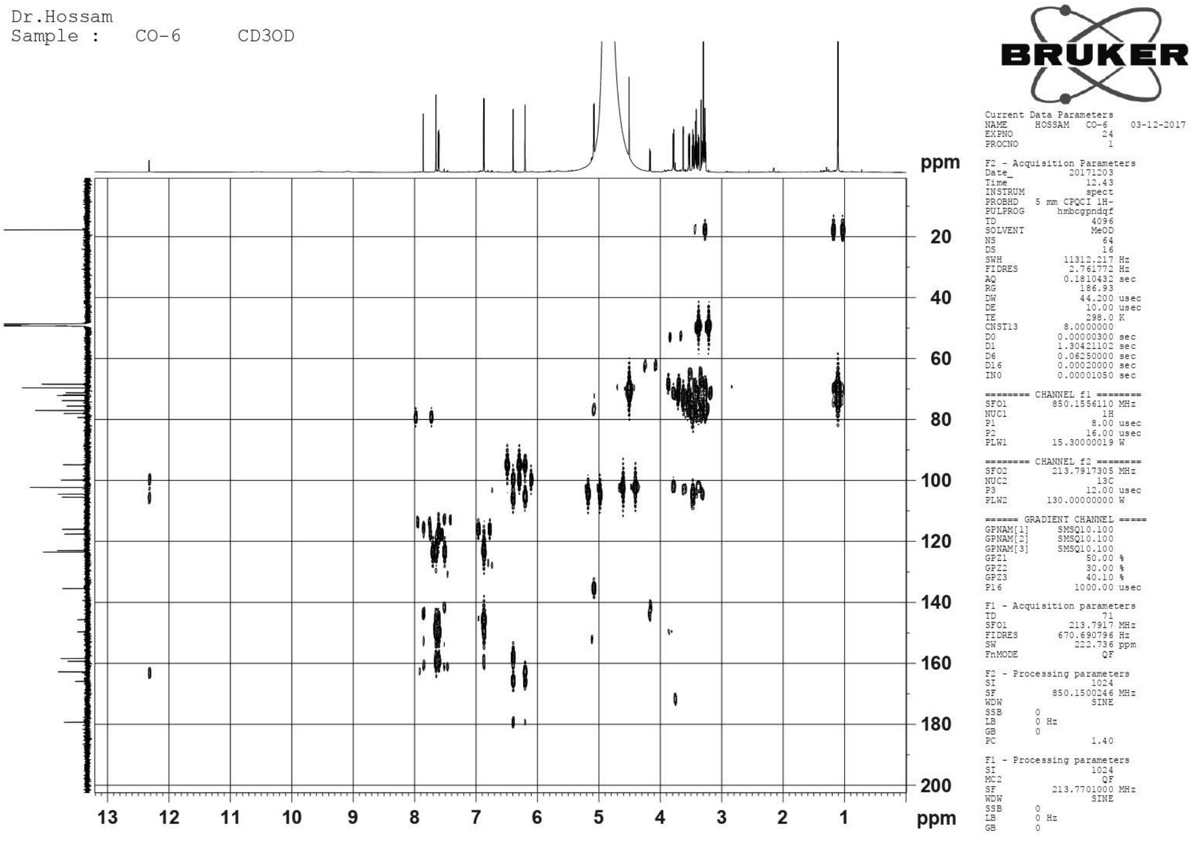

Figure S19. HMBC of compound **4** (DMSO, 850 and 213 MHz).

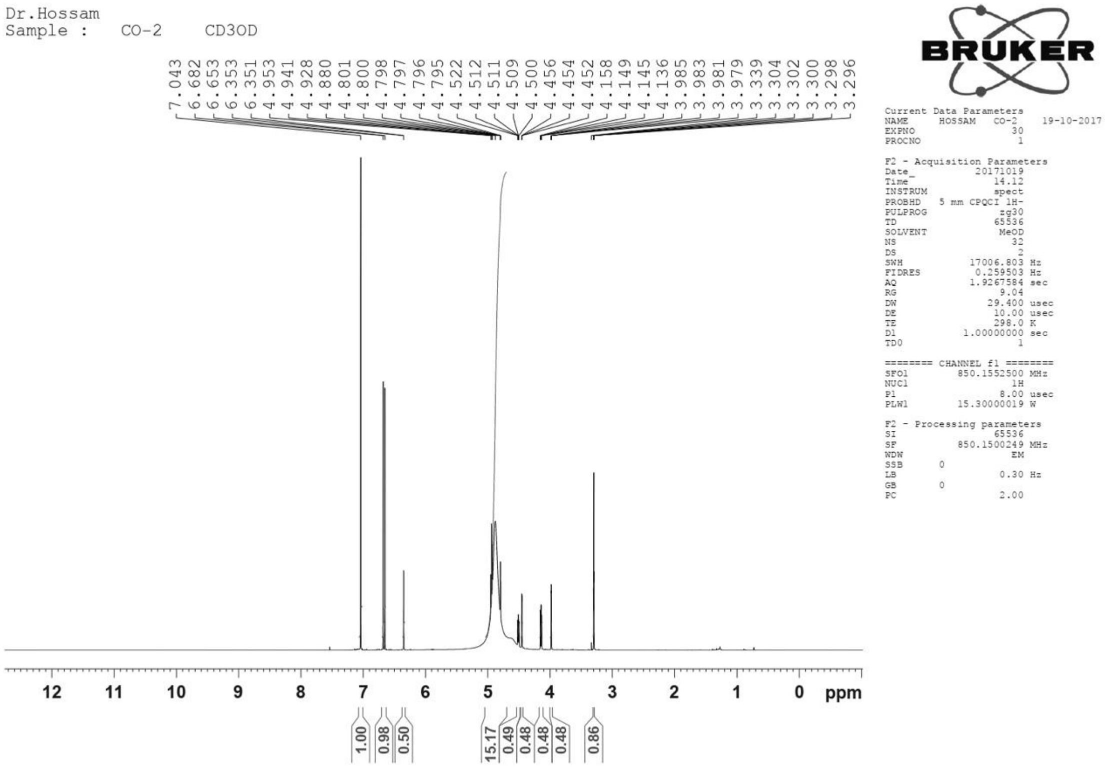

Figure S20. ¹HNMR of compound **5** (DMSO, 850 MHz).

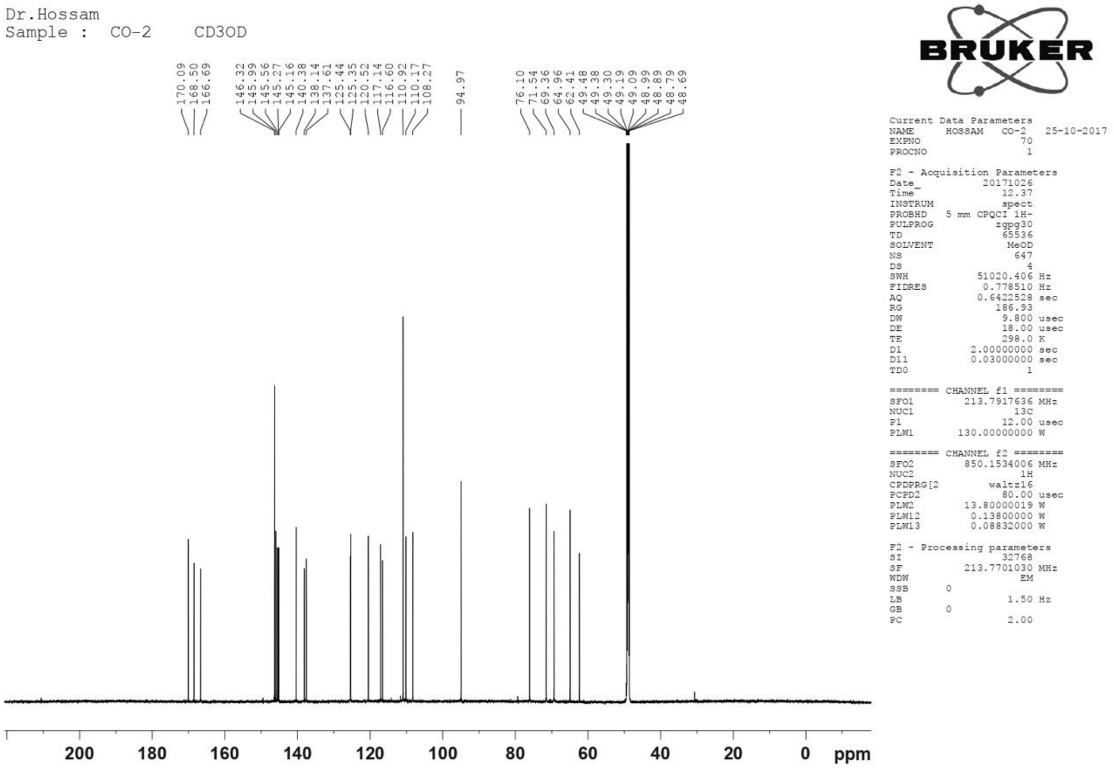

Figure S21. ^{13}C NMR of compound **5** (DMSO, 213 MHz).

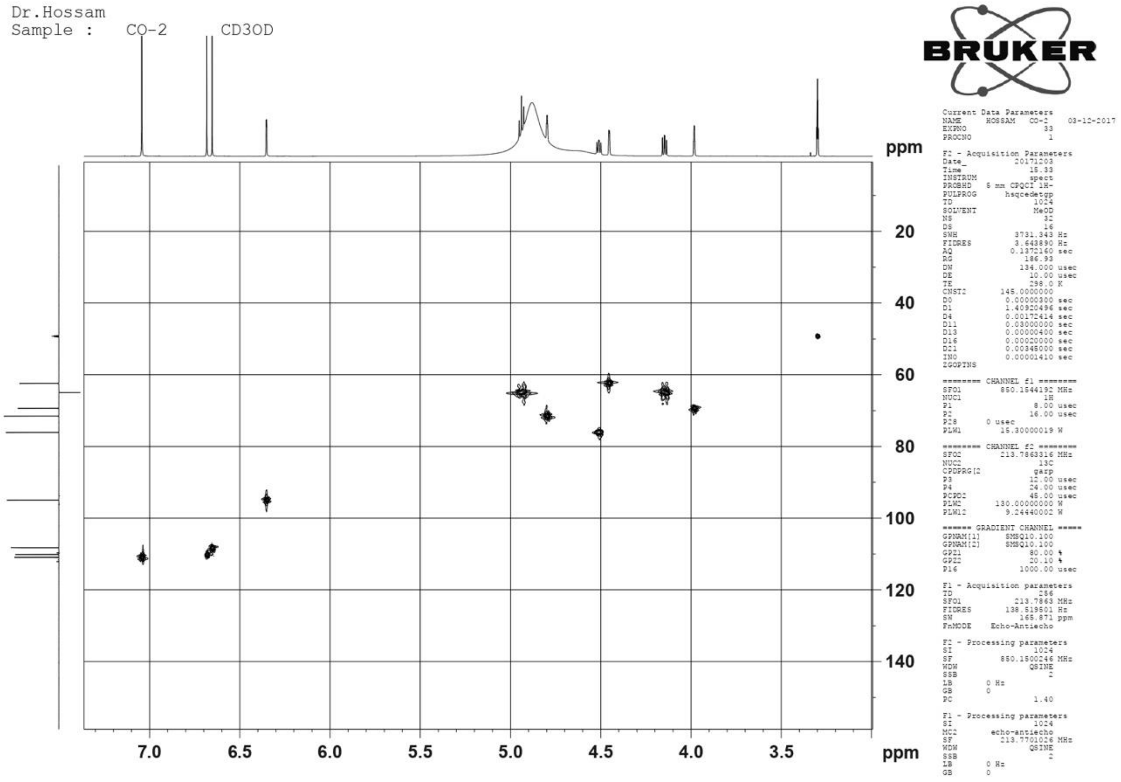

Figure S22. HSQC of compound **5** (DMSO, 850 and 213 MHz).

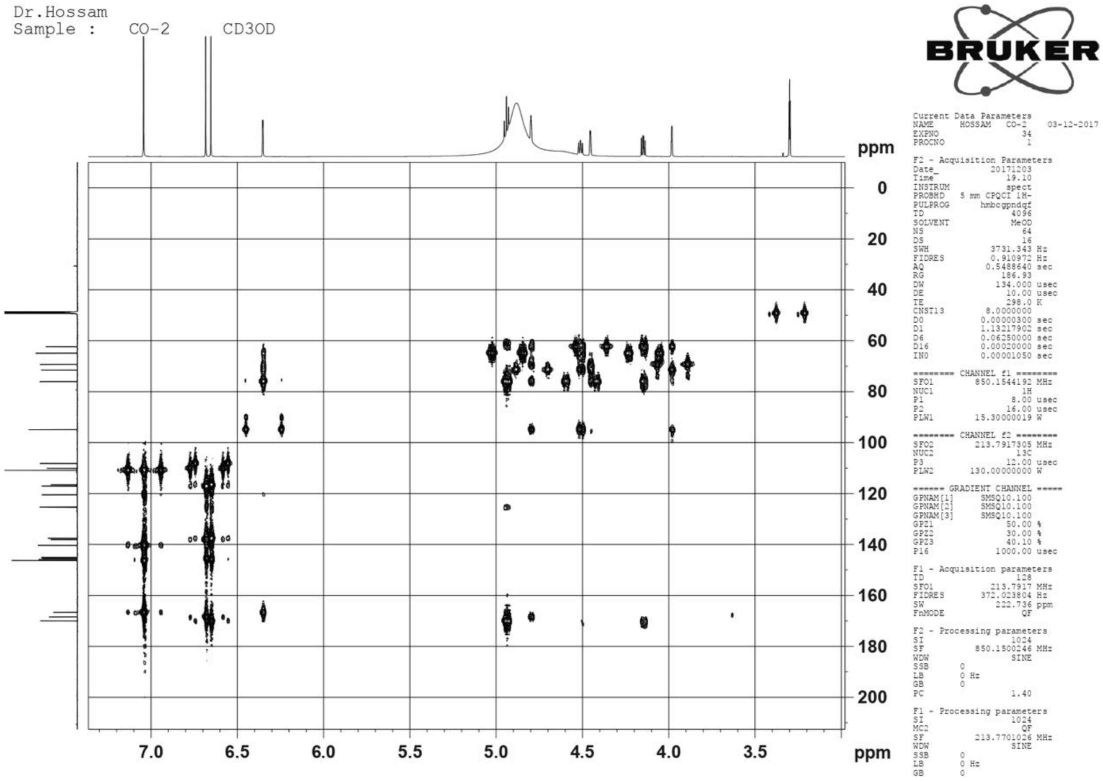

Figure S23. HMBC of compound **5** (DMSO, 850 and 213 MHz).
